# Epigenetic age and accelerated aging phenotypes: a tumor biomarker for predicting colorectal cancer

**DOI:** 10.18632/aging.206276

**Published:** 2025-07-07

**Authors:** Su Yon Jung, Matteo Pellegrini, Xianglong Tan, Herbert Yu

**Affiliations:** 1 Translational Sciences Section, School of Nursing, University of California, Los Angeles, CA 90095, USA; 2 Department of Epidemiology, Fielding School of Public Health, University of California, Los Angeles, CA 90095, USA; 3 Jonsson Comprehensive Cancer Center, University of California, Los Angeles, CA 90095, USA; 4 Department of Molecular, Cell and Developmental Biology, Life Sciences Division, University of California, Los Angeles, CA 90095, USA; 5 Department of Biological Chemistry, David Geffen School of Medicine, University of California, Los Angeles, CA 90095, USA; 6 Institute of Quantitative and Computational Biosciences, University of California, Los Angeles, CA 90095, USA; 7 Cancer Epidemiology Program, University of Hawaii Cancer Center, Honolulu, HI 96813, USA

**Keywords:** epigenetic aging, pre-diagnostic DNA, DNA methylation–based aging marker, colorectal cancer, carcinogenesis, oophorectomy, diet, postmenopausal women

## Abstract

Background: Epigenetic clocks, estimated via DNA methylation (DNAm), reflect individuals’ biological aging in multiple tissues and are associated with age-related diseases, but their functional role in colorectal cancer (CRC), an age-associated disease, remains unconclusive. DNAm in tumor tissues exclusively exhibits cancerization with expansion of a stem cell pool, leading to the lowest DNAm age; this raises a question about its cancer predictability. Thus, the DNAm aging marker in pre-diagnostic peripheral blood leukocytes (PBLs) may provide key information on CRC etiology and prevention. We aim to examine pre-diagnostic epigenetic makers for aging in PBLs in association with CRC development and risk modification by lifestyles.

Methods: Using data from a large cohort study of white postmenopausal women, we examined biological aging status in PBLs via three well-established epigenetic clocks—Horvath’s, Hannum’s and Levine’s—and prospectively evaluated CRC development in relation to the aging markers and risk modification by lifestyle factors.

Results: The epigenetic clocks strongly correlated with chronological age, and older DNAm age and age acceleration were significantly associated with increased risk for CRC. Women with bilateral oophorectomy before natural menopause had substantially higher risk for CRC development when they also had epigenetically accelerated aging phenotypes. Among women who maintained healthy dietary patterns, no apparently higher risk was found in those with accelerated aging compared with those with decelerated aging.

Conclusions: Our findings contribute to better understanding of the role of a pre-diagnostic epigenetic aging biomarker and its interplay with lifestyles in CRC carcinogenesis, informing risk stratification strategies for aged individuals.

## INTRODUCTION

Colorectal cancer (CRC) is the most common cancer and cause of cancer death in the U.S.A. and worldwide [1–4]. It is an age-associated disease: 90% of new cases develop in people aged 50 years and older [5, 6], emphasizing the role of age as an important predicting factor. However, the risk of CRC development substantially differs between the same aged individuals, highlighting heterogeneity in biological aging between people of the same age [7]. Thus, chronological age may not represent time-dependent biologic modifications well at the individual level.

Indeed, aging is viewed as a gradual decline in biological function, which correlates with various molecular alterations [8]. Lifelong exposures to harmful environments and risky behavioral factors may affect various biological aging processes at molecular levels, leading to cellular vulnerability, cell senescence, genomic and epigenomic instability, mitochondrial dysfunction, and telomere attrition [9]. Whereas genetic mutations in part explained individual differences in biological aging processes [10], alterations in DNA methylation (DNAm), a major epigenetic modification, are known to be the most accurate readout of aging, by capturing the independent genetic influence and its interaction with environment on molecular functions. Aging is thus considered a reduced stability of epigenetic marks and epigenetic deviation from chronological age [11, 12]. For example, a study of monozygotic twins [13] demonstrated that differences in DNAm variation at genome-wide and regional levels were greater in older than in younger twin pairs, suggesting that reduced stability of the epigenome is age related.

In addition, DNAm alteration is one of the distinct features in cancers, including CRC [14–16]. At the molecular level, CRC is largely attributable to the lifetime accumulation of genetic and epigenetic alterations in the colonic epithelium. In particular, abnormal DNAm changes over time often result in initiation of irregular stem and progenitor cell growth in the intestine, mediating field cancerization, and further induce the carcinogenetic process [16, 17]. Thus, the DNAm-based aging marker called “epigenetic” or “DNAm” age may better catch individuals’ susceptibility to CRC, an age-associated disease.

DNAm-based estimators of epigenetic age—epigenetic clocks—have been developed [18–20] and are known to reflect the effects of genetic and environmental factors and their interaction on cellular function across time. They are thus highly accurate markers of biological aging, strongly correlating with chronological age in multiple tissues [18, 21–23]. However, their functional roles in association with CRC are inconclusive, showing inconsistent findings across studies: positive [24–28] but also negative [7] and null [29] associations between DNAm-age markers and CRC outcomes. This can largely owe to the use of different study designs in capturing CRC risk (cross-sectional, retrospective, or prospective), various clocks, different tissue types (e.g., blood- vs. tissue-based DNAm), heterogeneous samples (e.g., different ages, sexes, and races), and different population-specific environmental and behavioral profiles.

Of note, DNAm in tumor tissues exhibits the state of cancerization in tumor cells, exclusively reflecting the capability of differentiation in malignant clones and expansion of a stem cell pool, leading to the lowest DNAm age [30]; this raises the question of its utility as a cancer predictor in a comparison study with normal tissues. On the other hand, given that cancers do not develop as an isolated phenomenon in their target tissues, and other organs are systemically involved in carcinogenesis through the immune and metabolic systems via the peripheral bloodstream [31], DNAm changes in peripheral blood leukocytes (PBLs) may reflect comprehensive carcinogenetic mechanisms by capturing key information about the epigenetic interplay with cumulative environmental and lifestyle factors that disrupt epigenetic balance and thus increase cancer susceptibility [32]. Therefore, a study of DNAm-age markers, specifically measured pre-diagnostically in PBLs, the tissue type most easily accessible from healthy people, has an important implication in CRC prediction and prevention. We addressed this need in our study by examining epigenetic aging markers estimated in pre-diagnostic PBLs in association with CRC development.

Interestingly, racial variation in age-drift pattern, or deviation from chronological age, is noted: greater DNAm age accel (age acceleration, defined as DNAm age exceeding chronological age) occurs in colorectal tissues among whites than in other races [33]. This highlights the need for a race-specific study on the epigenetic aging process in CRC. Our study thus focused on postmenopausal women, highly vulnerable to CRC, among whites, a majority study population.

We investigated various conventional CRC risk factors and prospective development of CRC in association with well-established epigenetic clocks measured in pre-diagnostic PBLs. We further examined how the cancer risk prediction associated with epigenetic aging differs by selected lifestyle factors. We performed a validation study using an independent CRC cohort with pre-diagnostic PBL-based DNAm data and additionally analyzed tissue-based DNAm data from two independent CRC cohorts for comparison. Our purpose was to detect a pre-diagnostic epigenetic aging marker in PBLs, an easily accessible and less invasively obtained tissue, taking into account the role of lifestyles, therefore better strategizing risk stratification for CRC development. This may further contribute to promotion of potential preventive strategies for those at high risk.

## MATERIALS AND METHODS

### Selection of study population

We obtained the PBL-based genome-wide DNAm data from a prospective cohort database, the Women’s Health Initiative Database for Genotypes and Phenotypes (WHI-dbGaP) genetic repository, consisting of postmenopausal women, 50–79 years old at their enrollment from 1993 to 1998 at >40 U.S.A. clinical centers [34–36]. From the dbGap database, we extracted available DNAm data from the BAA23 [37]. Since different races exhibit different patterns of DNAm age [38], we examined only non–Hispanic white women, a major subpopulation within this study (i.e., 998 whites of total 2,107). We included women with no cancer diagnosis at enrollment and with a follow-up period at least 1 year (to minimize reverse causality inference). This resulted in 955 women; 29 of them developed primary colorectal carcinoma during a mean 17-year follow-up.

To validate our findings, we obtained one independent dataset from the National Center for Biotechnology Information Gene Expression Omnibus (GEO) database (accession number GSE51032). This cohort’s participants had been enrolled between 1993 and 1998, and the European Prospective Investigation into Cancer and Nutrition (EPIC-Italy) study has generated global-level DNAm in PBLs through the Human Genetics Foundation in Turin, Italy [24, 39]; they were followed up for >15 years, containing 79 women with primary CRC development and 340 women without cancer. Additionally, we analyzed CRC tissue-based global-level DNAm data using two independent datasets from The Cancer Genomic Atlas (TCGA) COADREAD Study [40] and another GEO database (accession number GSE199057 [41]). For our study, we examined only white women from each dataset, resulting in 146 tissues (134 CRC and 12 normal adjacent colorectal tissues) from TCGA and 105 tissues (36 CRC, 35 normal adjacent, and 34 normal tissues from participants without CRC development) from GSE199057. Our study was approved by the institutional review boards of the WHI clinical centers and the University of California, Los Angeles.

### Collection of basic participant characteristics and CRC outcomes

Self-administered questionnaires were completed by women at the time of their enrollment. Information included their demographic factors (age, race, and ethnicity), morbidities (treatment of type 2 diabetes (T2DM)), behavioral factors (daily intake of whole fruits, vegetables, and fatty acids assessed by the Healthy Eating Index (HEI)-2015 [42]; alcohol consumption; years as a regular smoker; and physical activity), and reproductive factors (both ovary removal and exogenous estrogen (E) use, such as unopposed E-only and opposed E plus progestin (P) from pills or patches). Their anthropometric measurements (height, weight, and waist and hip circumferences) were obtained by trained staff at screening.

A committee of physicians reviewed the patients’ medical records and pathology/cytology reports and after adjudicating primary CRC development, coded into the WHI database according to the National Cancer Institute’s Surveillance, Epidemiology, and End-Results guidelines [43]. The time from enrollment until CRC development, censoring, or study end-point was estimated as the number of years.

The CRC tissue–based cohorts from TCGA and GSE199057 and the PBL-based cohort with primary CRC development from GSE51032 include participants’ information on age, sex, race, and diagnosed tumor type. With the two tissue-based cohorts, we analyzed data from primary colorectal adenocarcinoma tissues and normal tissues adjacent to CRC, and with the GEO data only, normal tissues from those who remained cancer-free.

### DNAm array and epigenetic clocks

Genome-wide DNAm array in the WHI participants was performed by using their PBL-based DNA samples via Illumina 450 BeadChip, beta-mixture quantile (BMIQ) normalization [44], and batch adjustment with plate and chip as random intercept and row as a fixed effect [45], resulting in 482,421 CpG dinucleotides (CpGs). To confirm stability of DNAm from stored samples [46], as suggested by Horvath’s methods [18], we estimated leukocyte heterogeneities and adjusted in calculating DNAm age scales for CD4^+^ T cells, natural killer cells, monocytes, and granulocytes (Houseman’s method [47]), and for plasma blasts, CD8^+^CD28^–^CD45RA^–^ T cells, and naïve CD8 T cells (Horvath’s method [18]).

Global-levels of DNAm were generated from PBLs in the GSE51032 cohort and from CRC tissues in both TCGA and GSE199057 cohorts by Illumina 450 BeadChip (GSE51032 and TCGA) and Illumina EPIC (GSE199057). Using *minfi*, the data were normalized via normal-exponential out-of-band (Noob) background correction [48], and batch effects were corrected using Bland Altman methods for replicate samples [41]. For GSE51032, DNAm age was generated by accounting for leukocyte heterogeneities.

The biological clock of aging was the measurement via predicting an individual’s chronological age and relevant phenotypes based on their DNAm level. We used three well-known epigenetic clocks, including two first-generation clocks (Horvath’s clock [18, 49], a pan-tissue predictor with 353 CpGs, and Hannum’s PBL-based clock, with 71 CpGs [19]), and one of the second-generation clocks [27] (Levine’s whole-blood–based clock, with 513 CpGs [20]). Among the various second-generation clocks, Levine’s clock employs the concept of phenotypic aging, using ten selected clinical phenotypes and produces a phenotypic age score, further validated with multiple large epigenetic studies. Also, it shows the race-specific DNAm age difference. The three clocks we selected, with distinct biological focuses in various tissues, complementarily supported our analyses. DNAm age is a composite scale of a linear combination of the weighted CpGs at the individual level. Each clock was calculated by an online tool [18, 49] and the *methylclock* annotation Bioconductor package.

### Statistical analysis

We calculated the deviation of epigenetic age from chronological age with two estimates: 1) AgeAccelDiff, the departure of DNAm age from chronological age, calculated by subtracting chronological age from DNAm age, and 2) IEAA (intrinsic epigenetic age acceleration), the residual from regressing DNAm age on chronological age, which further adjusts for different cell counts. The IEAA represents cell-intrinsic aging effects independently from the variations of DNAm levels due to heterogeneity in cell components between individuals [50].

With each epigenetic clock, we examined the relationship between DNAm age and the two epigenetic age-departure measures (AgeAccelDiff and IEAA) and chronological age via linear regression and Spearman’s rank correlation coefficient testing in all women combined and also by CRC status. The distributions of DNAm age and the two age-departure measures by traditional CRC risk factors were examined via independent samples *t* or one-way ANOVA tests when applicable. Wilcoxon’s rank-sum and Kruskal-Wallis methods were used as appropriate when the variables were not normally distributed. Further, DNAm age and the two age-departure estimates were regressed as continuous and binary outcomes on individual CRC risk variables in overall participants and by CRC status; this reflects a one-unit increase in the risk variable in relation to increase in DNAm age/age accel in units of years.

In each clock, differences in levels of DNAm age and the two age-departure estimates by CRC status were tested using independent samples *t* or Wilcoxon’s rank-sum tests as appropriate. Additionally, we split each of the two age-departure measures into two categories, age accel and age decel (age deceleration, defined as DNAm that falls behind age) for conducting the Kaplan-Meier analysis with a log-rank test. In a multiple Cox proportional hazards regression evaluating the relationship between DNAm age/age departure and CRC risk, we confirmed that an assumption test was met via a Schoenfeld residual plot and rho, and controlled for traditional CRC risk variables [51–54] such as age; body mass index (BMI); waist-to-hip ratio (WHR); T2DM; alcohol consumption; years as a regular smoker; physical activity; and daily fruit, vegetable, and fat intake assessed by HEI-2015; bilateral oophorectomy; and hormone replacement therapy. The hazard ratio (HR) refers to a 1-year older DNAm age and age accel in relation to an increased CRC risk. We additionally examined DNAm age and age accel for every 10-year increase. Additionally, the follow-up period was restricted by removing those with <5 years of follow-up to exclude the potential for reverse association. Given that our tested questions were derived from our hypothesis that biological aging is associated with CRC risk and traditional risk factors, a two-tailed *p* < 0.05 was considered statistically significant.

For the GSE51032 women, we conducted Cox regression for CRC development. In both TCGA and GSE199057 cohorts, we performed logistic regression for each clock in relation to CRC tissues, compared with normal adjacent CRC tissues, by restricting analyses within women to see whether the results were comparable to those in the WHI and GSE51032 populations. Using only data from the GSE199057 cohort, we additionally conducted analyses for epigenetic aging between CRC and normal tissues from participants who remained cancer free and compared the findings with those from analyses between CRC and normal adjacent CRC tissues.

Lastly, we conducted stratification analyses by selected CRC risk factors in the WHI participants and examined how the effects of biological aging markers on CRC risk differed according to the risk factors.

### Availability of data and materials

The data that support the findings of this study are available in accordance with policies developed by the NHLBI and WHI in order to protect sensitive participant information and approved by the Fred Hutchinson Cancer Research Center, which currently serves as the IRB of record for the WHI. Data requests may be made by emailing helpdesk@WHI.org.

## RESULTS

### Association of DNAm age, age accel, and IEAA with chronological age

**Figure 1:**
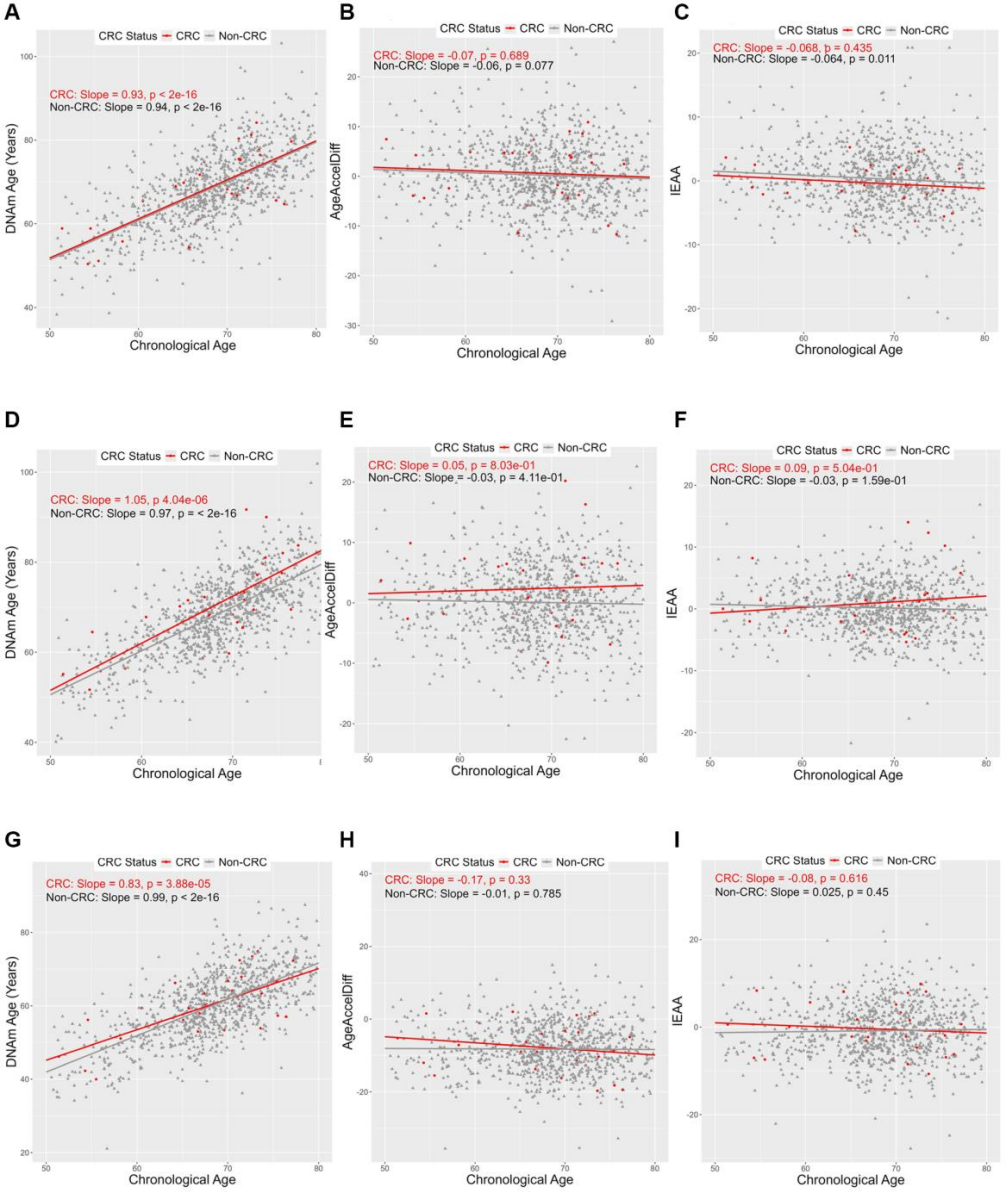
**Correlation between DNAmAge, AgeAccelDiff, and IEAA and chronological age by CRC status.** (AgeAccelDiff, epigenetic age acceleration as departure of DNAmAge from chronological age; CRC, colorectal cancer; DNAmAge, DNA methylation–based marker of aging; IEAA, intrinsic epigenetic age acceleration as residuals adjusted for cell composition). Horvath’s clock: (**A**) DNAmAge; (**B**) AgeAccelDiff; (**C**) IEAA. Hannum’s clock: (**D**) DNAmAge; (**E**) AgeAccelDiff; (**F**) IEAA. Levine’s clock: (**G**) DNAmAge; (**H**) AgeAccelDiff; (**I**) IEAA.

With all three biological clocks (Figure 1), a positive relationship between DNAm age and chronological age (termed simply age, hereafter) was observed in both women who developed CRC and those who did not, whereas AgeAccelDiff and IEAA showed no substantial association with age.

### Relationship between traditional CRC risk factors and biological aging markers

**Figure 2:**
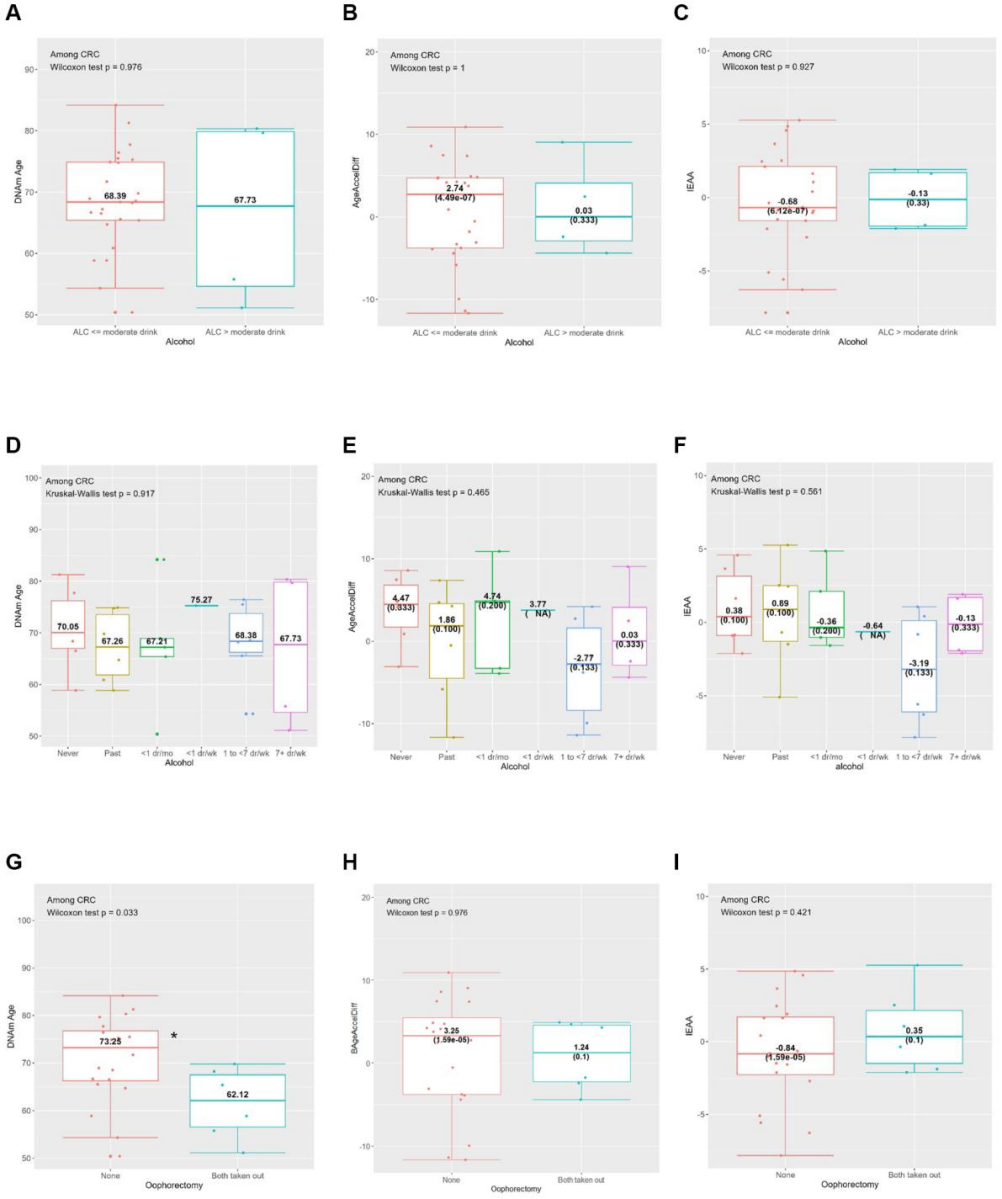
**Horvath’s clock: distribution of DNAmAge, AgeAccelDiff, and IEAA by selected CRC risk factors among CRC patients.** (AgeAccelDiff, epigenetic age acceleration as departure of DNAmAge from chronological age; CRC, colorectal cancer; DNAmAge, DNA methylation–based marker of aging; IEAA, intrinsic epigenetic age acceleration as residuals adjusted for cell composition). (**A**) Alcohol (binary): DNAmAge; (**B**) Alcohol (binary): AgeAccelDiff; (**C**) Alcohol (binary): IEAA; (**D**) Alcohol (6 categories): DNAmAge; (**E**) Alcohol (6 categories): AgeAccelDiff; (**F**) Alcohol (6 categories): IEAA; (**G**) Oophorectomy: DNAmAge; (**H**) Oophorectomy: AgeAccelDiff; (**I**) Oophorectomy: IEAA.

With Horvath’s clock (Tables 1–3 and Supplementary Table 1; Figure 2 and Supplementary Figure 1), multiple CRC risk factors demonstrated significant associations with the clock estimates in all women, combining those who developed CRC and those who stayed cancer free. With all three measures, including DNAm age and as both continuous and binary outcomes, AgeAccelDiff and IEAA, BMI had a dose-response relationship with an approximately 5-year older age and age accel increase among an extremely obese group (BMI >40), compared with a normal-weight group (BMI 18.5 to <25) (Tables 1–3 and Supplementary Figure 1A–1C). Similar positive patterns were observed between WHR and the three estimates for DNAm age and age departure (Supplementary Figure 1D–1F).

**Table 1:** Association of DNAmAge in Horvath’s clock with selected CRC risk factors^*^.

**CRC risk factor**	**Effect size**	**95% CI**	***P***
Age^**^	**0.94**	**(0.87, 1.00)**	**7.27E-12**
BMI^§^ (normal weight vs. underweight, BMI <18.5)	0.41	(−3.87, 4.69)	0.850
Overweight, BMI ≥25 and BMI <30	0.55	(−0.68, 1.78)	0.379
Obesity, BMI ≥30 and BMI <40	**1.42**	**(0.09, 2.76)**	**0.037**
Extreme obesity, BMI ≥40	**5.63**	**(2.70, 8.55)**	**0.0002**
Waist-to-hip ratio	**10.64**	**(3.39, 17.88)**	**0.004**
Waist-to-hip ratio^¥^ (≤0.85 vs. >0.85)	**1.50**	**(0.34, 2.66)**	**0.011**
Alcohol intake (never vs. past drinker)	−1.02	(−3.12, 1.07)	0.339
<1 drink per month	−1.72	(−3.89, 0.45)	0.120
<1 drink per week	−1.02	(−3.08, 1.04)	0.333
1 to <7 drinks per week	**−2.15**	**(−4.13, −0.17)**	**0.033**
7+ drinks per week	−2.16	(−4.43, 0.11)	0.062
Years of regular smoking (never vs. <5 years)	−0.04	(−1.93, 1.85)	0.969
5 to <20 years	−1.72	(−3.60, 0.16)	0.074
20 + years	**−1.86**	**(−3.28, −0.44)**	**0.010**
Healthy Eating Index-2015, whole fruits	**0.95**	**(0.55, 1.36)**	**4.14E-06**
Healthy Eating Index-2015, whole fruits^¥^ (≤4.10 vs. >4.10)	**2.37**	**(1.13, 3.62)**	**0.000**
Healthy Eating Index-2015, vegetables^¥^ (≤4.23 vs. >4.23)	**1.22**	**(0.07, 2.38)**	**0.038**
Oophorectomy history (never vs. both ovary removal)	**1.52**	**(0.02, 3.01)**	**0.048**
Exogenous estrogen only (never use vs. <5 years)	**2.34**	**(0.83, 3.86)**	**0.002**
5 to < 10 years	−1.39	(−4.07, 1.28)	0.307
10 + years	**2.67**	**(0.41, 4.92)**	**0.021**
Exogenous estrogen plus progestin (never use vs. <5 years)	**−3.38**	**(−5.78, −0.98)**	**0.006**
5 to <10 years	−3.02	(−7.72, 1.68)	0.208
10 + years	−3.04	(−8.33, 2.25)	0.260
**Only among CRC patients**
Oophorectomy history (never vs. both ovary removal)	**−9.29**	**(−17.55, −1.03)**	**0.029**

Abbreviations: BMI: body mass index; CI: confidence interval; CRC: colorectal cancer; DNAmAge: DNA methylation–based marker of aging. Numbers in bold face are statistically significant. ^*^Only factors having *statistically significant* association with DNAmAge are displayed. ^**^Age was further significant in a multiple regression model, adjusting for covariates (age, BMI, waist-to-hip ratio, type 2 diabetes, oophorectomy history, hormone replacement therapy, diet including whole fruits, vegetables, and fatty acids from Healthy Eating Index-2015, alcohol intake, years of regular smoking, and physical activity (except tested variable(s))). ^§^Variables were significant only in a multiple regression model. ^¥^Waist-to-hip ratio was categorized using 0.85 as the cutoff, at which higher values fall into the viscerally obese range [84]; Healthy Eating Index-2015, whole fruits and vegetables, were dichotomized by the mean, 4.10, and the median, 4.23, respectively.

**Table 2:** Association of AgeAccelDiff in Horvath’s clock with selected CRC risk factors^*^.

**CRC risk factor**	**Effect size**	**95% CI**	***P***
BMI	**0.17**	**(0.10, 0.24)**	**8.02E-06**
BMI^**^ (normal weight vs. underweight, BMI <18.5)	0.05	(−4.14, 4.24)	0.981
Overweight, BMI ≥25 and BMI <30	0.75	(−0.33, 1.83)	0.175
Obesity, BMI ≥30 and BMI <40	**2.05**	**(0.97, 3.13)**	**0.0002**
Extreme obesity, BMI ≥40	**5.75**	**(3.28, 8.22)**	**5.64E-06**
Waist-to-hip ratio	**7.43**	**(1.96, 12.89)**	**0.008**
Waist-to-hip ratio^¥^ (≤0.85 vs. >0.85)	**1.07**	**(0.19, 1.94)**	**0.017**
Physical activity^¥^ (< 10 MET vs. ≥10 MET)	**−0.92**	**(−1.82, −0.02)**	**0.045**

Abbreviations: AgeAccelDiff; epigenetic age acceleration measured as departure of DNAmAge from chronological age; BMI: body mass index; CI: confidence interval; CRC: colorectal cancer; MET: metabolic equivalent. Numbers in bold face are statistically significant. ^*^Only factors having *statistically significant* association with DNAmAge are displayed. ^**^BMI was further significant in a multiple regression model, adjusting for covariates (age, BMI, waist-to-hip ratio, type 2 diabetes, oophorectomy history, hormone replacement therapy, diet including whole fruits, vegetables, and fatty acids from Healthy Eating Index-2015, alcohol intake, years of regular smoking, and physical activity (except tested variable(s))). ^¥^Waist-to-hip ratio was categorized using 0.85 as the cutoff, at which higher values fall into the viscerally obese range [84]; Physical activity was estimated from recreational physical activity records combining walking and mild, moderate, and strenuous physical activity. Each activity was assigned a MET value corresponding to intensity and the total MET·hours·week per week was stratified into two groups, with 10 METs as the cutoff according to current American College of Sports Medicine and American Heart Association recommendations [63].

**Table 3:** Association of IEAA in Horvath’s clock with selected CRC risk factors^*^.

**CRC risk factor**	**Effect size**	**95% CI**	***P***
BMI	**0.10**	**(0.04, 0.15)**	**0.000**
BMI^**^ (normal weight vs. underweight, BMI <18.5)	0.17	(−2.85, 3.18)	0.914
Overweight, BMI ≥25 and BMI <30	0.50	(−0.28, 1.28)	0.207
Obesity, BMI ≥30 and BMI <40	**1.14**	**(0.37, 1.92)**	**0.004**
Extreme obesity, BMI ≥40	**3.49**	**(1.71, 5.26)**	**0.0001**
Waist-to-hip ratio	**4.14**	**(0.23, 8.05)**	**0.038**
Healthy Eating Index-2015, vegetables^§¥^ (≤4.23 vs. >4.23)	**0.78**	**(0.06, 1.49)**	**0.033**
**Only among CRC patients**			
Alcohol intake (never vs. past drinker)	−0.50	(−4.29, 3.29)	0.786
<1 drink per month	−0.20	(−4.17, 3.78)	0.919
<1 drink per week	−1.64	(−8.73, 5.45)	0.636
1 to <7 drinks per week	**−4.17**	**(−7.96, −0.38)**	**0.033**
7+ drinks per week	−1.11	(−5.35, 3.13)	0.592

Abbreviations: BMI: body mass index; CI: confidence interval; CRC: colorectal cancer; IEAA: intrinsic epigenetic age acceleration as residuals adjusted for cell composition. Numbers in bold face are statistically significant. ^*^Only factors having *statistically significant* association with DNAmAge are displayed. ^**^BMI was further significant in a multiple regression model, adjusting for covariates (age, BMI, waist-to-hip ratio, type 2 diabetes, oophorectomy history, hormone replacement therapy, diet including whole fruits, vegetables, and fatty acids from Healthy Eating Index-2015, alcohol intake, years of regular smoking, and physical activity (except tested variable(s))). ^§^Variable was significant only in a multiple regression model. ^¥^Healthy Eating Index-2015, vegetables, was dichotomized by the median, 4.23.

Compared with never drinkers, frequent drinkers (1 to <7 drinks/week), and those with greater than moderate alcohol intake (>14 g/day) than the counterpart were associated with younger DNAm age and decreased age accel in the IEAA (Tables 1–3 and Supplementary Table 1; Supplementary Figure 1G–1L). This pattern was also observed in relation to the IEAA when analysis was restricted to women who developed CRC (Figure 2A–2F). Similarly, longer-term regular smokers (≥20 years), compared with never smokers, had younger DNAm age by about 2 years (Table 1 and Supplementary Figure 1M). Whereas a greater intake of whole fruits and vegetables were associated with increased aging accel in DNAm age and IEAA, the opposite—a decelerated aging trend—was observed in AgeAccelDiff, despite insufficient statistical power (Supplementary Figure 1P–1U). As expected, the physically active group (≥10 MET) had about a 1-year decreased age accel measured by AgeAccelDiff (Table 2 and Supplementary Figure 1Y–1AA).

In relation to women’s reproductive history, women with both ovary removal had about a 2-year-older DNAm age than women with both ovaries intact; however, the opposite pattern was observed when the analysis was restricted to women who developed CRC: younger DNAm age in those with both ovary removal (Table 1, Figure 2G–2I and Supplementary Figure 1BB–1DD). Interestingly, unopposed E-only users had a fluctuating but generally increased pattern of DNAm age than never users, with older age in short-term (<5 years) and the longest-term (≥10 years) users but a slightly though nonsignificant younger age in the medium-term (5 to <10 years) users. In contrast, deceased DNAm age was observed in opposed E plus P users. The short-term (<5 years) users had younger DNAm age and a similar younger aging pattern was shown for longer-term users. (Table 1 and Supplementary Figure 1EE–1JJ).

In Hannum’s clock (Supplementary Table 2 and Supplementary Figure 2), the observed patterns were consistent with those from Horvath’s clock, specifically for BMI, WHR, exercise, and E-only and E plus P users. Similarly, greater alcohol intake and more years of regular smoking were associated with younger DNAm age. However, these patterns differed in women who developed CRC: older DNAm age and the accelerated age accel of AgeAccelDiff and IEAA, particularly among those who consumed <1 drink/week and regular smokers for <5 years, compared with their respective never users (Supplementary Table 2 and Supplementary Figure 2J–2L, 2P–2R). In addition, greater intake of whole fruits was associated with decelerated IEAA (Supplementary Table 2 and Supplementary Figure 2U). Further, those with bilateral oophorectomy were associated, although not significantly, with increased age accel in AgeAccelDiff and IEAA (Supplementary Figure 2EE–2GG).

Levine’s clock demonstrated patterns generally matched to those found with the other two clocks across all CRC risk factors (Supplementary Table 3 and Supplementary Figure 3). As with Hannum’s clock, greater intake of whole fruits was associated with decreased age accel in AgeAccelDiff and IEAA. Of note, only Levine’s clock identified an association of T2DM with significantly older DNAm age and higher age accel measured from AgeAccelDiff.

### DNAm age and epigenetic age departure with prospective development of CRC

All three clocks’ patterns for the association with CRC development were consistent during a 15-year follow-up (Supplementary Table 4 and Supplementary Figure 4). A 1-year-older DNAm age was related to about 10% higher risk for CRC development; and this magnitude was much more profound with Horvath’s and Hannum’s clocks when analyzed as a 10-year interval: every 10-year-older DNAm age was associated with approximately four times increased risk for developing CRC. We confirmed this pattern by restricting the analysis to women who were followed at least for 5 years to exclude a potential reverse association. Likewise, when the AgeAccelDiff was categorized into ACC (accelerated age, i.e., DNAm age’s positive deviation from age) and DCC (decelerated age, i.e., DNAm age’s negative deviation from age), women with ACC on all three clocks had shorter cancer-free intervals and about 5–10 times higher risk for CRC development than did those with DCC. In only Levine’s clock, those with ACC in IEAA were associated with a three-times-greater risk for CRC.

### Biological aging markers in association with CRC in PBLs (GSE 51032) and in tissues (TCGA and GSE 199057)

In the GSE51032 women (Supplementary Table 5 and Supplementary Figure 5), older DNAm age in PBLs was observed in those who developed CRC than in those who remained cancer free, a pattern similar to that found in the WHI. An analysis of two tissue-based datasets, TCGA (Supplementary Table 6 and Supplementary Figure 6) and GSE199057 (Supplementary Table 7 and Supplementary Figure 7) on women, displayed results similar to each other’s and also to those from the PBL-based datasets from GSE 51032 and WHI. In particular, Levine’s clock was positively associated with CRC tissues compared with adjacent normal tissues in both TCGA and GSE199057 datasets and with normal tissues from women who remained cancer free in GSE199057. Hannum’s clock revealed a positive association with CRC tissues, compared with adjacent normal tissues, only in the GSE199057 dataset.

### Stratification analyses by selected risk factors for biological aging markers and CRC development

**Figure 3:**
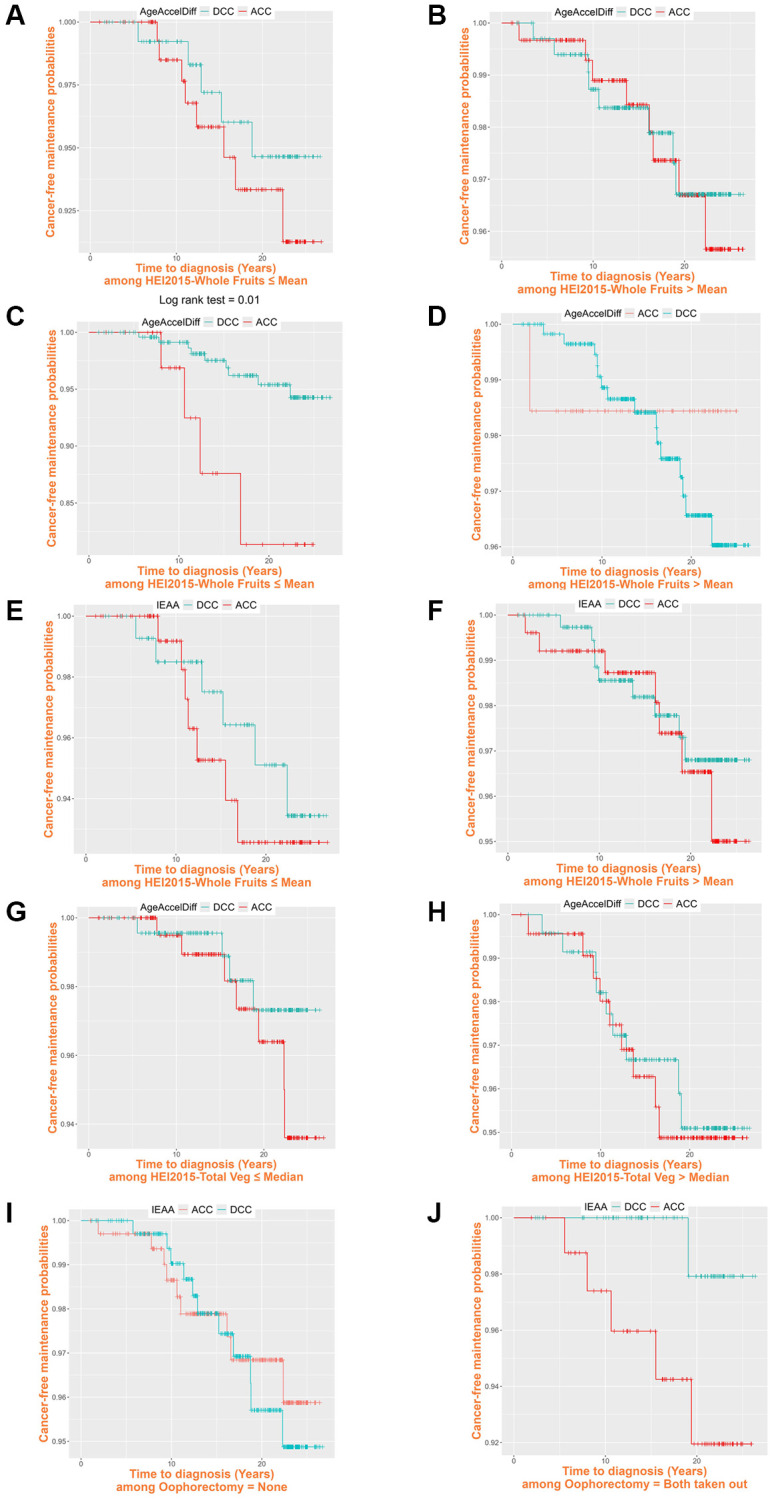
**Cancer-free probability curve of biological age by Healthy Eating Index-2015 whole fruit and vegetable intake and oophorectomy.** (ACC, accelerated age (positive deviation of DNAm age from age); AgeAccelDiff, epigenetic age acceleration as departure of DNAmAge from chronological age; DCC, decelerated age (negative deviation of DNAm age from age); IEAA, intrinsic epigenetic age acceleration as residuals adjusted for cell composition). Healthy Eating Index-2015 whole fruits: (**A**) ≤ Mean, 4.10: Horvath’s clock, AgeAccelDiff; (**B**) > Mean, 4.10: Horvath’s clock, AgeAccelDiff; (**C**) ≤ Mean, 4.10: Levine’s clock, AgeAccelDiff; (**D**) > Mean, 4.10: Levine’s clock, AgeAccelDiff; (**E**) ≤ Mean, 4.10: Levine’s clock, IEAA; (**F**) > Mean, 4.10: Levine’s clock, IEAA. Healthy Eating Index-2015 vegetables: (**G**) ≤ Median, 4.23: Horvath’s clock, AgeAccelDiff; (**H**) > Median, 4.23: Horvath’s clock, AgeAccelDiff. Oophorectomy: (**I**) Never: Hannum’s clock, IEAA; (**J**) Both ovary removal: Hannum’s clock, IEAA.

Given that greater intake of whole fruits and vegetables lowers the risk of CRC development [53] and that, in our analyses, it was generally associated with decreased age accel, we next examined how this greater intake affected CRC risk in relation to biological aging during follow-up (Table 4 and Figure 3). In Horvath’s and Levine’s clocks, among women with less intake of whole fruits, those with ACC in AgeAccelDiff than those with DCC and every 10-year age accel increase in IEAA had a six and 18 times higher risk for CRC development, respectively. Similarly, those who consumed fewer vegetables were associated with a more-than-20-times increased risk for developing CRC for every 10-year increase measured by age accel in Horvath’s AgeAccelDiff. However, for those who consumed more whole fruits and vegetables, no association between aging markers and CRC risk was found, indicating that the risk for CRC development in biologically older women is not higher than in younger women if they ate more than an average amount of whole fruits and vegetables (Figure 3A–3H). Also, considering that both ovary removal was associated with increased biological aging, we evaluated the effect of oophorectomy status on increased risk for CRC as they are biologically older. Whereas women with both ovaries intact had no higher risk for CRC with increased age accel, a 1-year age accel increase in Hannum’s IEAA was associated with >20 times higher risk for CRC development when women took out both ovaries (Figure 3I, 3J).

**Table 4:** Biological aging in association with CRC development, stratified by Healthy Eating Index-2015 whole fruit and vegetable intake and oophorectomy.

**DNAm Age clock**	**HR^†^**	**95% CI**	***P***	**HR^†^**	**95% CI**	***P***
**Healthy Eating Index-2015 whole fruits**						
**Horvath’s clock**		**≤ mean, 4.10**			**> mean, 4.10**	
AgeAccelDiff^*^	**1.16**	**(1.02, 1.31)**	**0.025**	1.01	(0.91, 1.11)	0.917
**Levine’s clock**						
AgeAccelDiff^*^	**1.19**	**(1.06, 1.32)**	**0.002**	0.97	(0.89, 1.05)	0.426
AgeAccelDiff, 10-year interval	**3.19**	**(1.26, 8.12)**	**0.015**	0.91	(0.42, 1.96)	0.808
AgeAccelDiff, ACC vs. DCC	**6.16**	**(1.22, 31.09)**	**0.028**	0.33	(0.03, 3.68)	0.367
IEAA^*^	**1.23**	**(1.07, 1.41)**	**0.003**	0.97	(0.89, 1.07)	0.560
IEAA, 10-year interval	**18.56**	**(5.66, 60.88)**	**1.00E-06**	0.88	(0.37, 2.12)	0.780
**Healthy Eating Index-2015 vegetables**						
**Horvath’s clock**		**≤ median, 4.23**			**> median, 4.23**	
AgeAccelDiff, 10-year interval	**26.72**	**(4.40, 162.33)**	**0.0004**	0.91	(0.37, 2.20)	0.832
**Oophorectomy**						
**Hannum’s clock**		**Never**			**Both ovary removal**	
AgeAccelDiff^*^	**1.09**	**(1.02, 1.16)**	**0.012**	**1.95**	**(1.68, 2.27)**	**<2e-16**
AgeAccelDiff, 10-year interval	1.39	(0.64, 3.00)	0.403	**12.91**	**(3.43, 48.61)**	**0.0001**
IEAA^*^	1.04	(0.92, 1.18)	0.524	**27.09**	**(19.82, 37.01)**	**<2e-16**

Abbreviations: ACC: accelerated age (positive deviation of DNAm age from chronological age); AgeAccelDiff: epigenetic age acceleration measured as departure of DNAmAge from chronological age; CI: confidence interval; CRC: colorectal cancer; DCC: decelerated age (negative deviation of DNAm age from chronological age); DNAmAge: DNA methylation–based marker of aging; HR: hazard ratio; IEAA: intrinsic epigenetic age acceleration as residuals adjusted for cell composition. Numbers in bold face are statistically significant. ^*^DNAmAge/AgeAccelDiff/IEAA were each analyzed as a continuous variable. ^†^HR adjusted for all covariates (age, body mass index, waist-to-hip ratio, type 2 diabetes, oophorectomy history, hormone replacement therapy, diet including whole fruits, vegetables, and fatty acids from Healthy Eating Index-2015, alcohol intake, years of regular smoking, and physical activity (except tested variable(s))).

## DISCUSSION

Despite multiple studies on CRC risk in elderly people, variation in CRC prediction continues even after accounting for chronological age and conventional CRC risk factors. This underscores a need for better understanding of the physiologic and systemic dysregulations in biological aging and the interplay with lifestyle exposures, which could provide an important clue to accurately predicting the risk for CRC for an individual. We addressed this need among postmenopausal women aged 50 years and older by estimating their biological aging and prospectively investigating CRC development and risk modification by lifestyles. We noted that DNAm is deeply involved in the process of aging, as represented by modifications in various molecular pathways displayed at different individual rates. [21, 55], and accumulation of aberrant DNAm is frequently found in aging tissues including blood [21, 56] and the colon [57, 58], dysregulating the genome expression and disrupting cell homeostasis. Thus, DNAm changes can play a key role as both driver and passenger in tumorigenic events. We estimated individuals’ biological age via DNAm-based epigenetic aging markers (i.e., epigenetic clocks) in pre-diagnostic PBLs and found that all three epigenetic clocks tested were strongly associated with the range of ages that we studied. Also, older DNAm age and accelerated aging drift were significantly related to prospective CRC risk, consistent with reports from previous studies [26, 59]. Our findings were consistent even after removing women with <5 years of follow-up, confirming that our interpretation was less likely affected by reverse causation inference. Also, given that the results were apparent after controlling for comprehensive CRC lifestyle factors, CRC evolution in the methylome was not substantially confounded by the lifestyles.

Owing to the multifaceted nature of CRC, many lifestyle factors are involved in CRC development, including obesity, obesity-associated diseases, alcohol intake, smoking, and dietary patterns [53, 60–62]. We found that overall and abdominal adiposity were related to older age measured in DNAm and increased age accel in a dose-response fashion and that being physically active was associated with decreased age accel, corroborating the current American College of Sports Medicine and American Heart Association recommendations [63, 64].

Also, a growing body of methylome-based aging studies on lifestyles confirmed a lower rate of biological aging with adherence to healthy lifestyles, reflecting the recommendations of current dietary guidelines [65]. For instance, alcohol intake and cigarette smoking increased age accel in blood [66, 67], which is generally in line with our findings, particularly in Hannum’s clock among those who developed CRC. Specifically, relatively less frequent alcohol intake (<1 drink/week) was associated with accelerated aging, indicating that a small amount of drinking can promote an adverse effect. Interestingly, moderate alcohol consumption (1 to <7 drinks/week for women [68]) was associated with decreased aging accel; this agrees with previous findings [69] that moderate alcohol intake is protective against cardiovascular disease (CVD), warranting a larger independent validation study.

We found that greater intake of whole fruits and vegetables lowered epigenetic aging accel, supported by a previous study [70] reporting that a high blood level of carotenoid, a surrogate indicator of greater intake of fruits and vegetables, is associated with decelerated aging. This anti-aging effect may act on the inflammatory and cardiometabolic systems, leading to protection against aging-associated diseases, such as CVD [71], stroke [72], and T2DM [73]. In our study, those dietary factors significantly had modification effect on CRC development in relation to aging accel, with three to 20 times greater risk of CRC for every 10-year increase in age accel among those with lower than average intake. Notably, no higher cancer risk was apparently observed in those with aging acceleration than in those with aging deceleration when they had greater intake. This suggests that consuming whole fruits and vegetables above a certain level (about 0.3 cup/1,000 kcal and 0.9 cup/1,000 kcal, respectively, both of which are closely aligned with the dietary guideline for clinical relevance [65]) is crucial for protecting against CRC development, particularly among people who have accelerated aging phenotypes. If this finding is validated, the epigenetic age could thus be an informative marker for targeting dietary interventions against CRC risk.

We also found that both ovary removal was associated with older DNAm age and increased aging accel. This is supported by findings from both population [74, 75] and *in vivo* studies [76–79] that an increased risk for death and CRC development is associated with the functional loss of ovaries before natural menopause. In particular, two *in vivo* studies [78, 79] demonstrated that 17β-estradiol deficiency in ovariectomized female mice increased intestinal tumors. This suggests a protective effect of estrogen on CRC tumorigenesis through ERβ that leads to anti-inflammatory and anti-proliferative mechanisms [80, 81]. Complete removal of the ovaries may synergistically exert its effect on CRC risk in combination with other factors that affect DNA repair systems and detoxification processes. [82]. Our study participants with both ovary removal experienced loss of ovaries before their natural menopause and had a substantial risk for developing CRC when they had epigenetically accelerated aging phenotypes. This has an important clinical implication about the role of epigenetic aging markers in identifying an early-risk group who may benefit from intensive screening for their CRC prevention.

One tissue-based cohort we studied performed an EPIC array, differing from the other cohorts that conducted an HM450K array. However, we confirmed that the missing CpGs on the EPIC did not substantially affect the accuracy of the epigenetic age estimation [83] and also that the sensitivity testing with common CpGs across the two arrays yielded nearly identical results [7]. Our validation PBL-based and other tissue-based datasets do not contain an extensive set of covariates, so the findings are not confirmatory and may not be directly compared. Our data lacked clinical information, such as CRC molecular subtypes and location, reducing our ability to control for several cancer characteristics that can be associated with the CRC methylome. With a limited CRC sample size, our study lacks statistical power. In particular, multiple combinations of lifestyle factors in the subgroup analyses resulted in several extreme ranges of risk magnitude. We thus caution about potential false positives, acknowledging that an independent large replication study is warranted. We also acknowledge the restricted generalizability of our findings to other populations. However, our study has strong enough merit to promote research on epigenetically informed decision making and to provide tailored cancer-preventive interventions. Considering the unique environmental nature of the colon, including the gut microbiome and digestion products, development of a colon-specific aging clock that integrates such cumulative microbiome and diet effects is warranted.

## CONCLUSIONS

In summary, women with epigenetically older age and accelerated aging phenotypes had increased risk for CRC development, and the risk was notably higher in women who underwent premature menopause because of oophorectomy, whereas no apparent risk was observed in women with a healthy diet. Our findings contribute to better understanding of the role of epigenetic aging markers in combination with risk lifestyles in CRC carcinogenesis, informing risk stratification and potential intervention strategies tailored to aged individuals with a high risk for CRC.

## Supplementary Materials

Supplementary Figures

Supplementary Tables
