# Supplementary material for: Epigenetic age and accelerated aging phenotypes: a tumor biomarker for predicting colorectal cancer
Source: Aging (Albany NY). 2025 Jul 7;17(7):1624–66. doi: 10.18632/aging.206276 (PMC12339025; doi:10.18632/aging.206276)
Supplement: Supplementary Figures [file aging-17-206276-s001.pdf]

SUPPLEMENTARY FIGURES

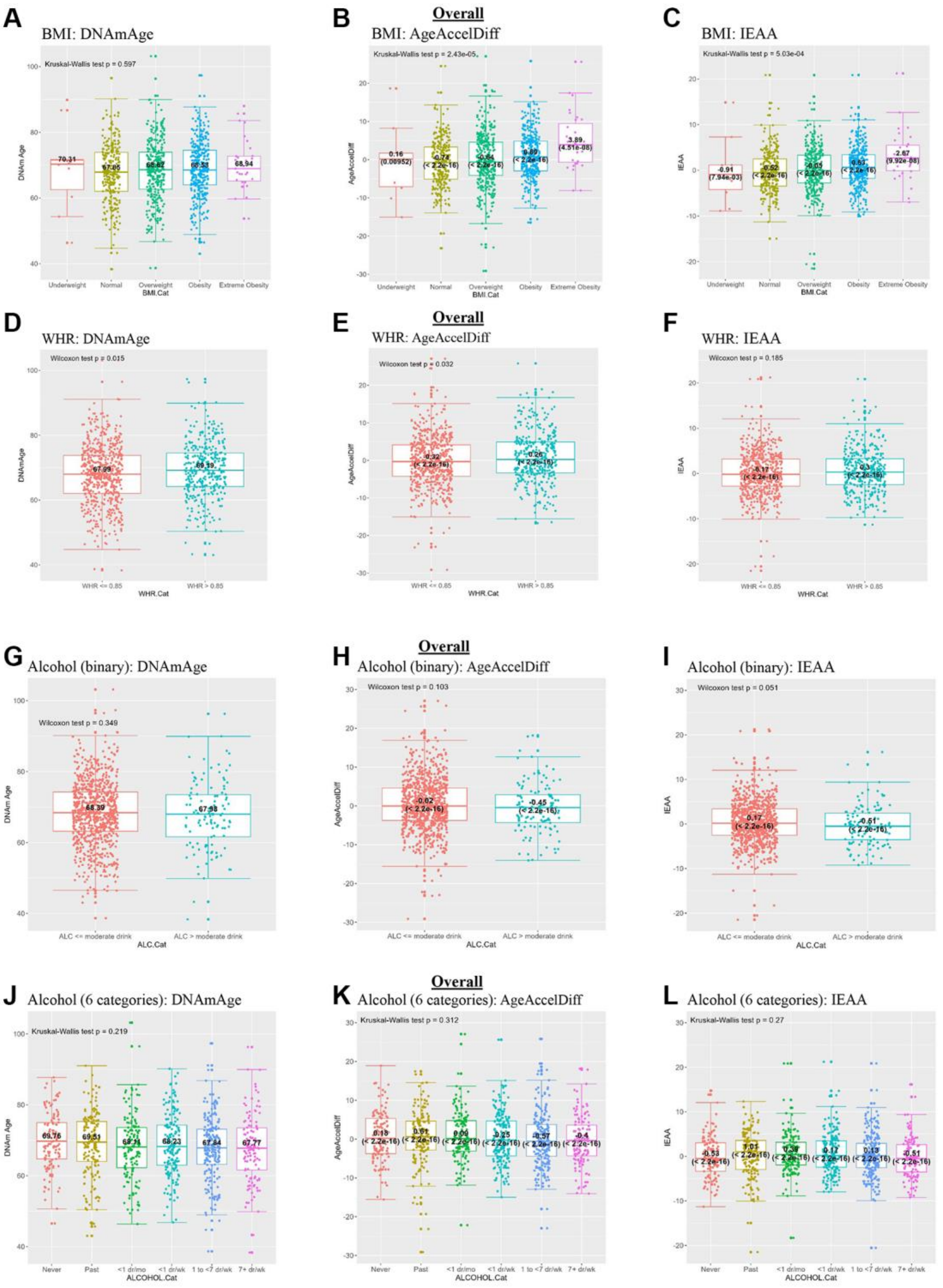

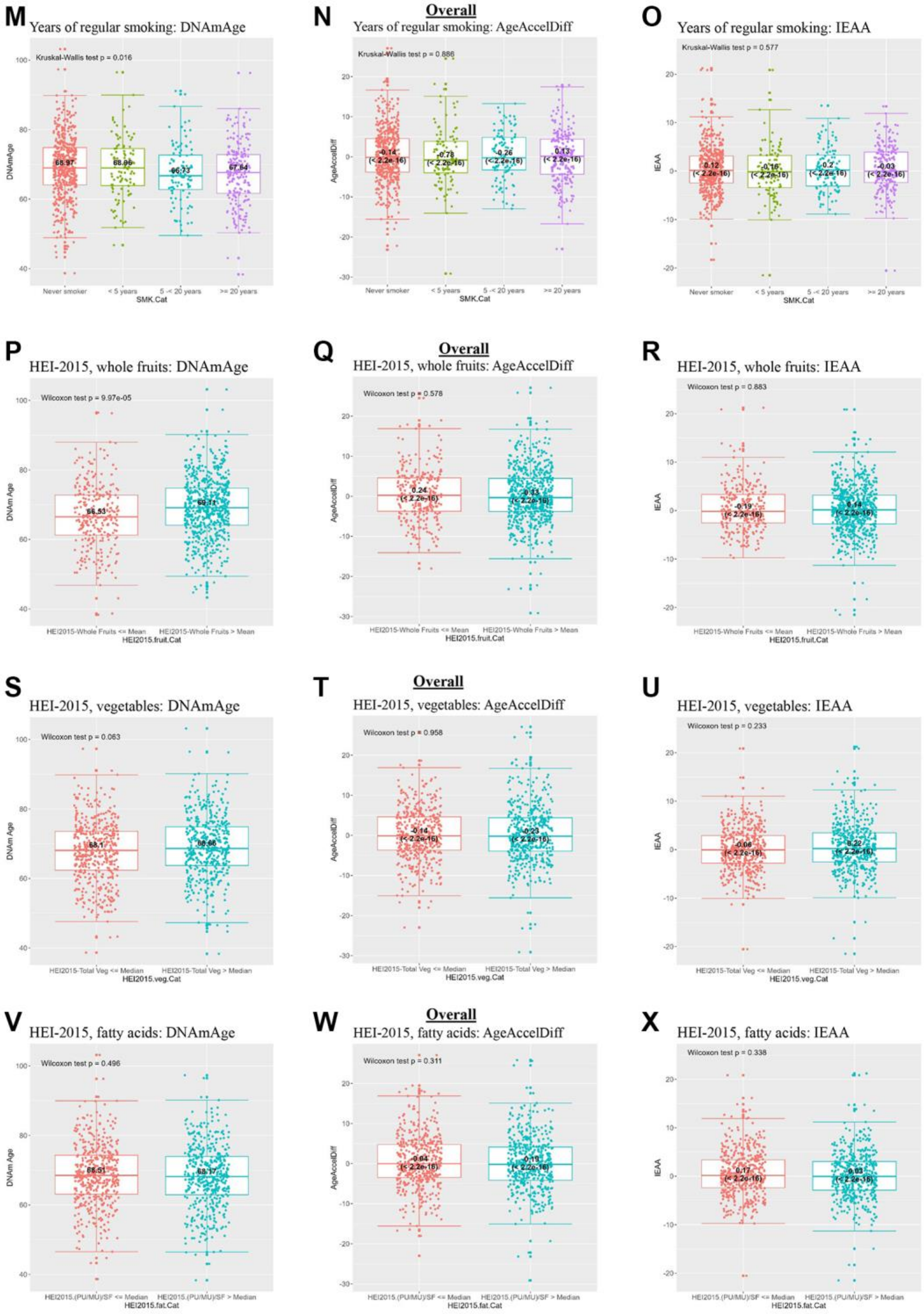
M
Years of regular smoking: DNAmAge
Kruskal-Wallis test p = 0.016
DNAmAge
Never smoker
< 5 years
5 -< 20 years
>= 20 years
SMK.Cat
N
Overall
Years of regular smoking: AgeAccelDiff
Kruskal-Wallis test p = 0.886
AgeAccelDiff
SMK.Cat
O
Years of regular smoking: IEAA
Kruskal-Wallis test p = 0.577
IEAA
SMK.Cat
P
HEI-2015, whole fruits: DNAmAge
Wilcoxon test p = 9.97e-05
DNAm Age
HEI2015-Whole Fruits <= Mean
HEI2015-Whole Fruits > Mean
HEI2015.fruit.Cat
Q
Overall
HEI-2015, whole fruits: AgeAccelDiff
Wilcoxon test p = 0.578
AgeAccelDiff
HEI2015.fruit.Cat
R
HEI-2015, whole fruits: IEAA
Wilcoxon test p = 0.883
IEAA
HEI2015.fruit.Cat
S
HEI-2015, vegetables: DNAmAge
Wilcoxon test p = 0.063
DNAm Age
HEI2015-Total Veg <= Median
HEI2015-Total Veg > Median
HEI2015.veg.Cat
T
Overall
HEI-2015, vegetables: AgeAccelDiff
Wilcoxon test p = 0.958
AgeAccelDiff
HEI2015.veg.Cat
U
HEI-2015, vegetables: IEAA
Wilcoxon test p = 0.233
IEAA
HEI2015.veg.Cat
V
HEI-2015, fatty acids: DNAmAge
Wilcoxon test p = 0.496
DNAm Age
HEI2015.(PU/MU)/SF <= Median
HEI2015.(PU/MU)/SF > Median
HEI2015.fat.Cat
W
Overall
HEI-2015, fatty acids: AgeAccelDiff
Wilcoxon test p = 0.311
AgeAccelDiff
HEI2015.fat.Cat
X
HEI-2015, fatty acids: IEAA
Wilcoxon test p = 0.338
IEAA
HEI2015.fat.Cat

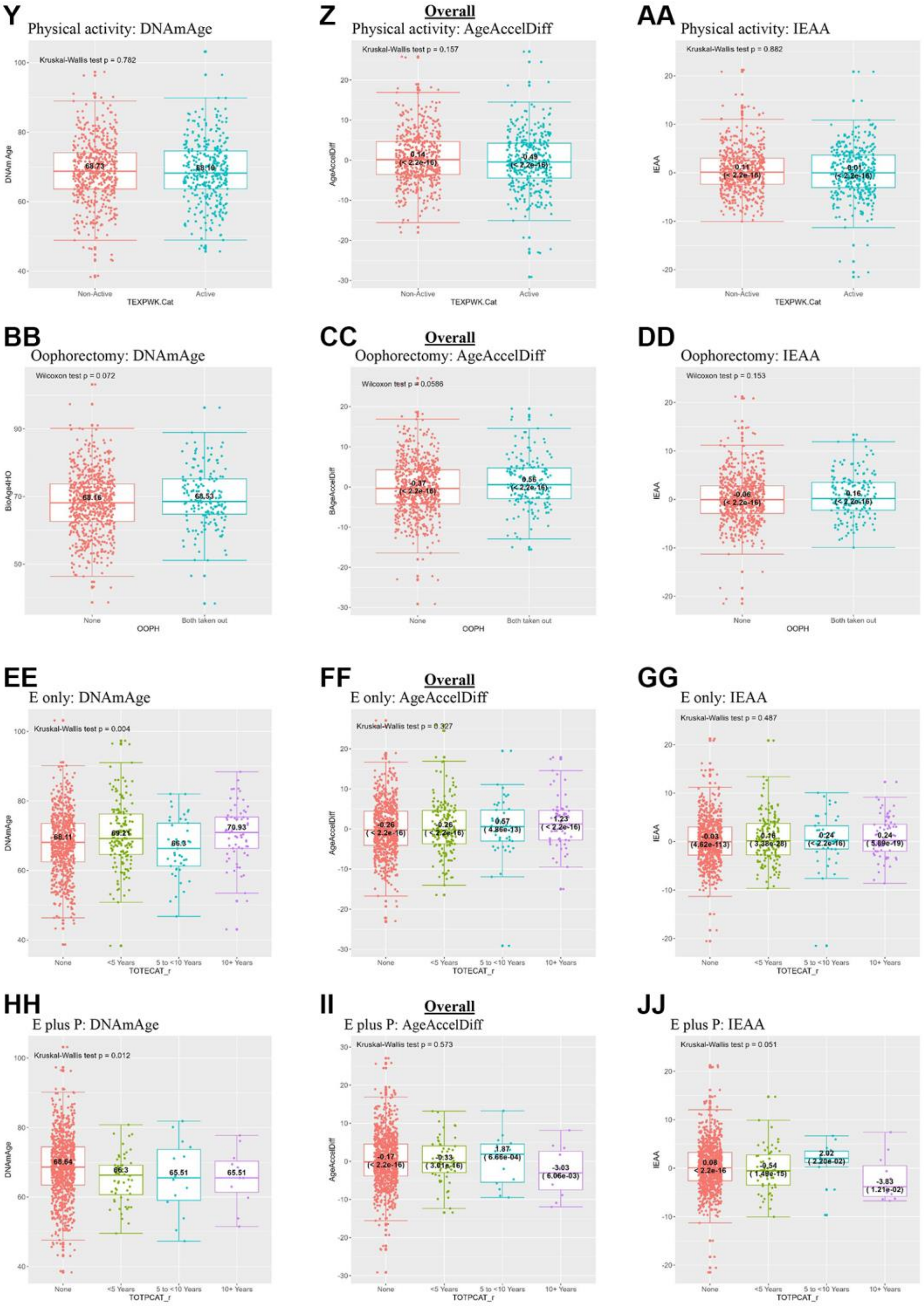

Supplementary Figure 1. Horvath's clock: distribution of DNAmAge, AgeAccelDiff, and IEAA by selected CRC risk factors in overall participants. Abbreviations: AgeAccelDiff: epigenetic age acceleration as departure of DNAmAge from chronologic age; BMI: body mass index; CRC: colorectal cancer; DNAmAge: DNA methylation-based marker of aging; E only: exogenous estrogen only; E plus P: E plus progestin; HEI-2015: Healthy Eating Index-2015; IEAA: intrinsic epigenetic age acceleration as residuals adjusted for cell composition; WHR: waist-to-hip ratio.

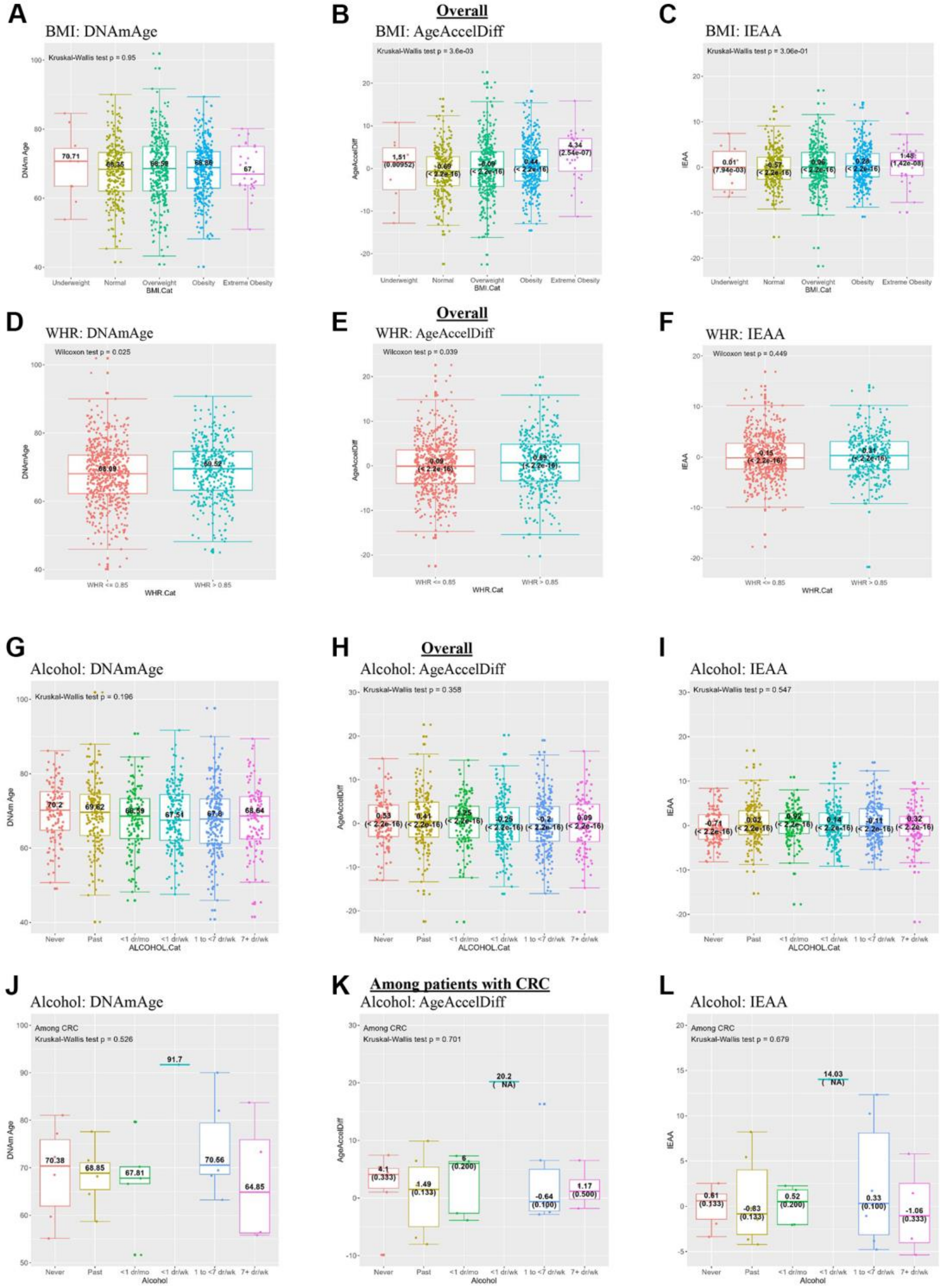
A
BMI: DNAmAge
Kruskal-Wallis test p = 0.95
B
Overall
BMI: AgeAccelDiff
Kruskal-Wallis test p = 3.6e-03
C
BMI: IEAA
Kruskal-Wallis test p = 3.06e-01
Underweight
Normal
Overweight
Obesity
Extreme Obesity
BMI.Cat
D
WHR: DNAmAge
Wilcoxon test p = 0.025
E
Overall
WHR: AgeAccelDiff
Wilcoxon test p = 0.039
F
WHR: IEAA
Wilcoxon test p = 0.449
WHR <= 0.85
WHR > 0.85
WHR.Cat
G
Alcohol: DNAmAge
Kruskal-Wallis test p = 0.196
H
Overall
Alcohol: AgeAccelDiff
Kruskal-Wallis test p = 0.358
I
Alcohol: IEAA
Kruskal-Wallis test p = 0.547
Never
Past
<1 dr/mo
<1 dr/wk
1 to <7 dr/wk
7+ dr/wk
ALCOHOL.Cat
J
Alcohol: DNAmAge
Among CRC
Kruskal-Wallis test p = 0.526
K
Among patients with CRC
Alcohol: AgeAccelDiff
Among CRC
Kruskal-Wallis test p = 0.701
L
Alcohol: IEAA
Among CRC
Kruskal-Wallis test p = 0.679
Alcohol

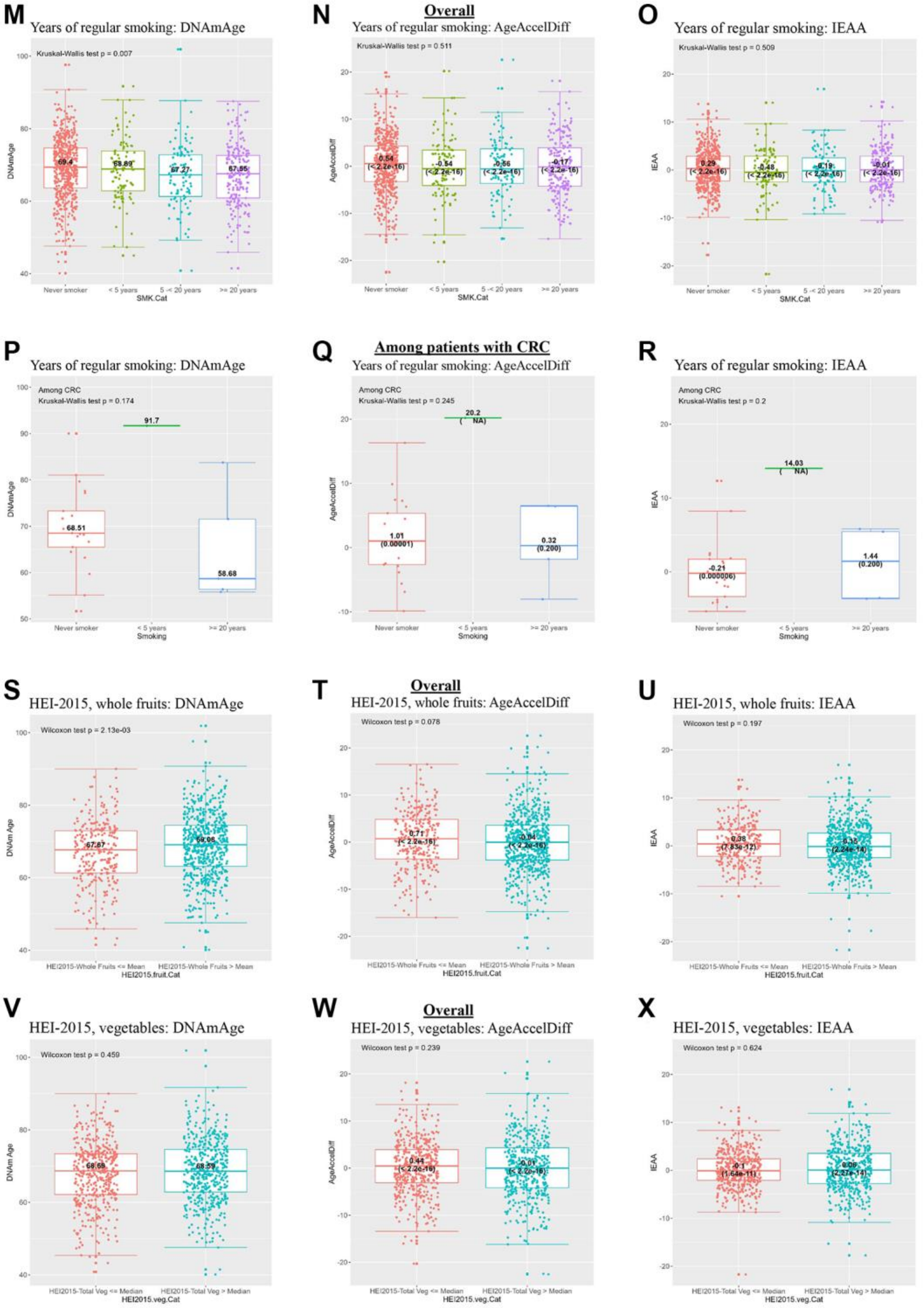
M
Years of regular smoking: DNAmAge
Kruskal-Wallis test p = 0.007
DNAmAge
SMK.Cat
Never smoker
< 5 years
5 -< 20 years
>= 20 years
N
Overall
Years of regular smoking: AgeAccelDiff
Kruskal-Wallis test p = 0.511
AgeAccelDiff
O
Years of regular smoking: IEAA
Kruskal-Wallis test p = 0.509
IEAA
P
Years of regular smoking: DNAmAge
Among CRC
Kruskal-Wallis test p = 0.174
91.7
68.51
58.68
Smoking
Q
Among patients with CRC
Years of regular smoking: AgeAccelDiff
Among CRC
Kruskal-Wallis test p = 0.245
20.2
1.01
(0.00001)
0.32
(0.200)
R
Years of regular smoking: IEAA
Among CRC
Kruskal-Wallis test p = 0.2
14.03
-0.21
(0.000006)
1.44
(0.200)
S
HEI-2015, whole fruits: DNAmAge
Wilcoxon test p = 2.13e-03
DNAm Age
HEI2015-Whole Fruits <= Mean
HEI2015-Whole Fruits > Mean
HEI2015.fruit.Cat
T
Overall
HEI-2015, whole fruits: AgeAccelDiff
Wilcoxon test p = 0.078
U
HEI-2015, whole fruits: IEAA
Wilcoxon test p = 0.197
V
HEI-2015, vegetables: DNAmAge
Wilcoxon test p = 0.459
HEI2015-Total Veg <= Median
HEI2015-Total Veg > Median
HEI2015.veg.Cat
W
Overall
HEI-2015, vegetables: AgeAccelDiff
Wilcoxon test p = 0.239
X
HEI-2015, vegetables: IEAA
Wilcoxon test p = 0.624

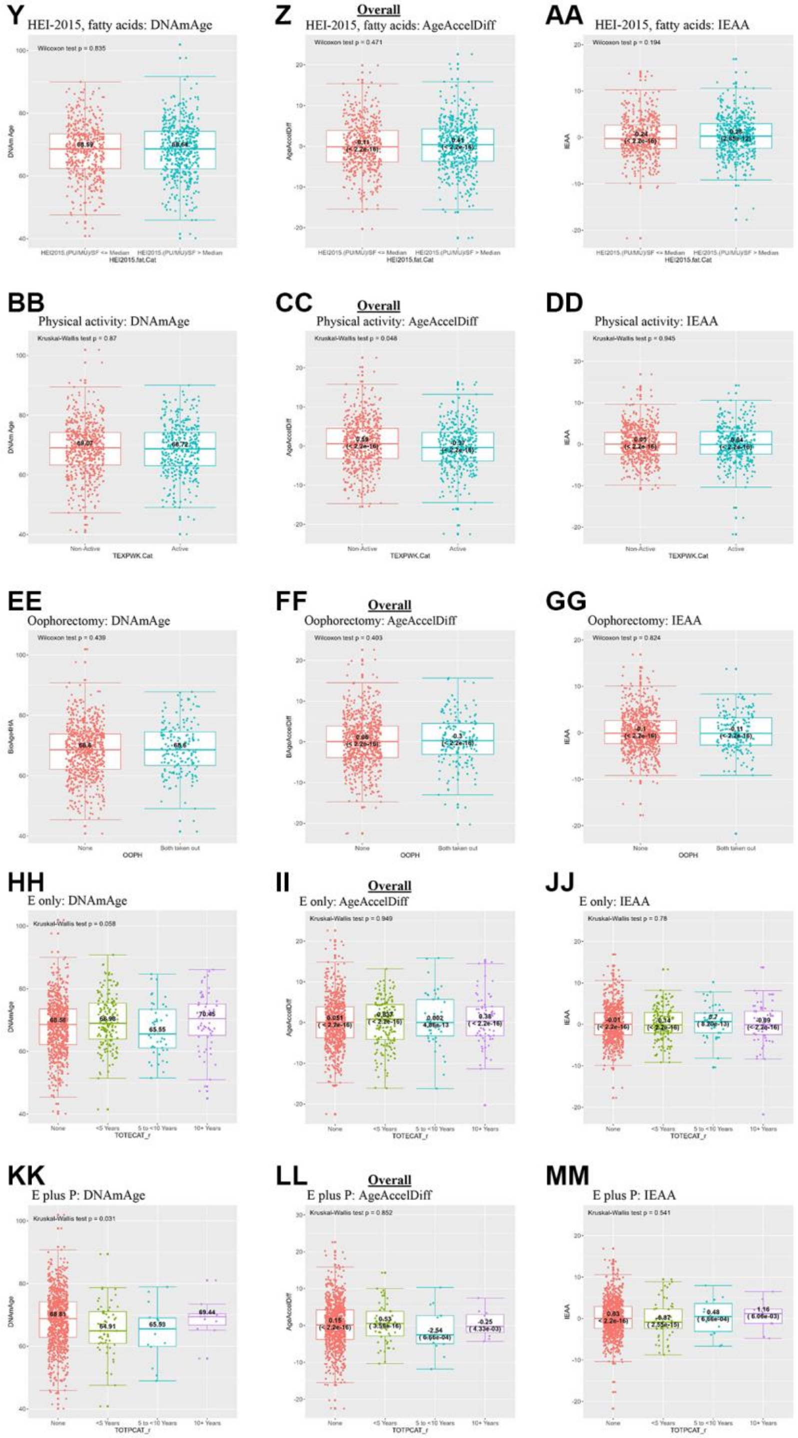

Supplementary Figure 2. Hannum's clock: distribution of DNAmAge, AgeAccelDiff, and IEAA by selected CRC risk factors. Abbreviations: AgeAccelDiff: epigenetic age acceleration as departure of DNAmAge from chronologic age; BMI: body mass index; CRC: colorectal cancer; DNAmAge: DNA methylationbased marker of aging; E only: exogenous estrogen only; E plus P: E plus progestin; HEI-2015: Healthy Eating Index-2015; IEAA: intrinsic epigenetic age acceleration as residuals adjusted for cell composition; WHR: waist-to-hip ratio.

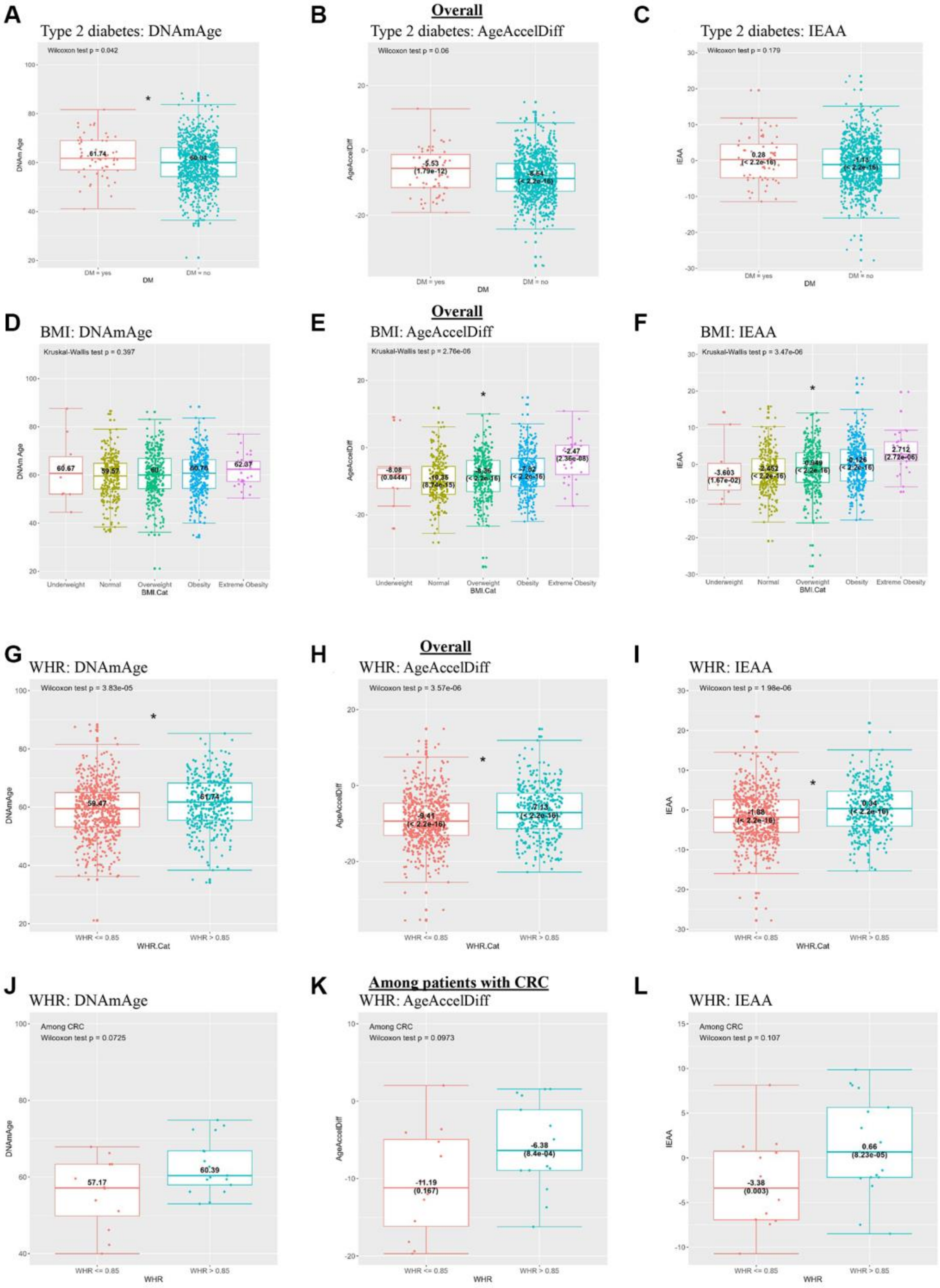

A
Type 2 diabetes: DNAmAge
Wilcoxon test p = 0.042
B
Overall
Type 2 diabetes: AgeAccelDiff
Wilcoxon test p = 0.06
C
Type 2 diabetes: IEAA
Wilcoxon test p = 0.179
D
BMI: DNAmAge
Kruskal-Wallis test p = 0.397
E
Overall
BMI: AgeAccelDiff
Kruskal-Wallis test p = 2.76e-06
F
BMI: IEAA
Kruskal-Wallis test p = 3.47e-06
G
WHR: DNAmAge
Wilcoxon test p = 3.83e-05
H
Overall
WHR: AgeAccelDiff
Wilcoxon test p = 3.57e-06
I
WHR: IEAA
Wilcoxon test p = 1.98e-06
J
WHR: DNAmAge
Among CRC
Wilcoxon test p = 0.0725
K
Among patients with CRC
WHR: AgeAccelDiff
Among CRC
Wilcoxon test p = 0.0973
L
WHR: IEAA
Among CRC
Wilcoxon test p = 0.107

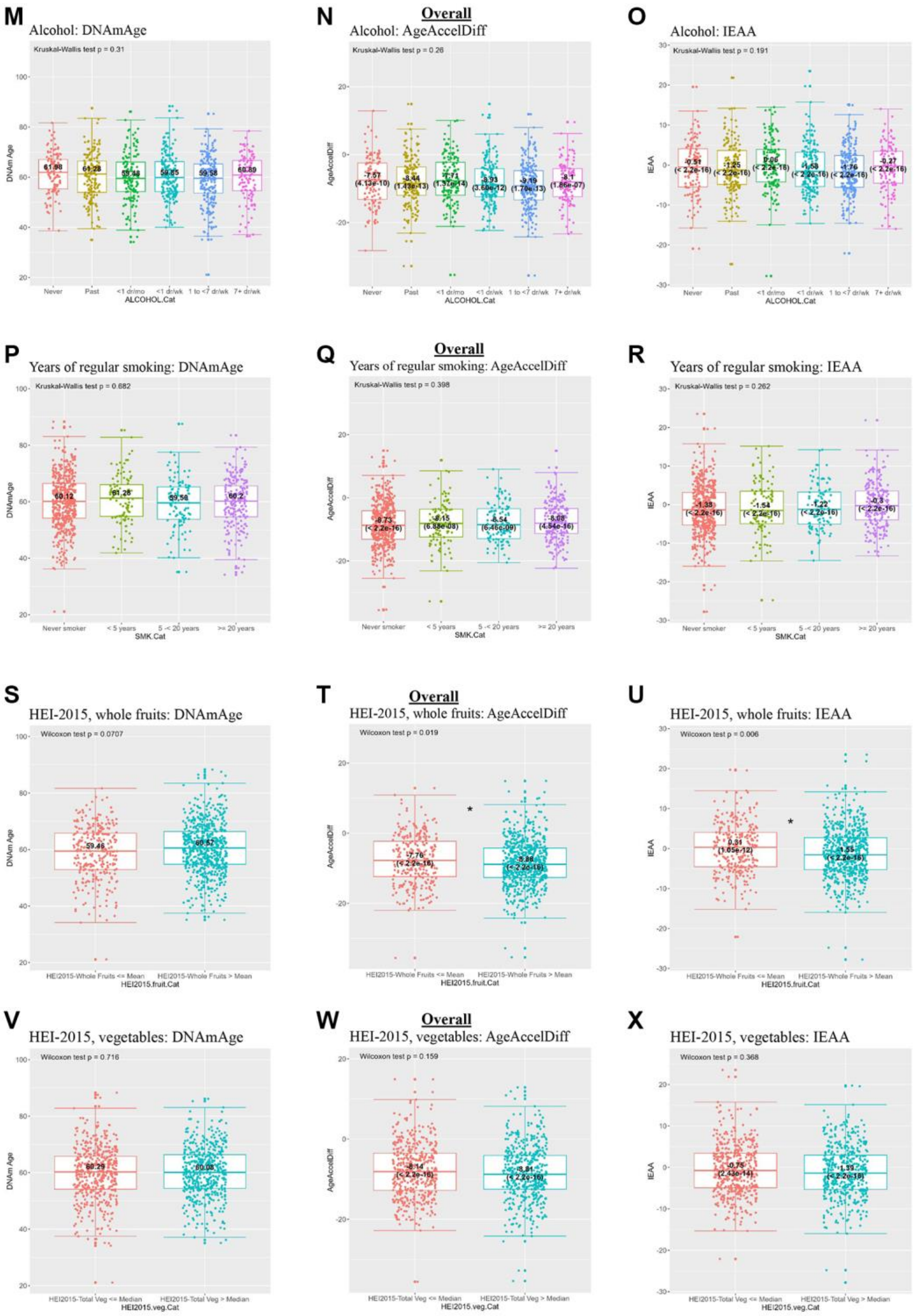
M
Alcohol: DNAmAge
Kruskal-Wallis test p = 0.31
N
Overall
Alcohol: AgeAccelDiff
Kruskal-Wallis test p = 0.26
O
Alcohol: IEAA
Kruskal-Wallis test p = 0.191
ALCOHOL.Cat
P
Years of regular smoking: DNAmAge
Kruskal-Wallis test p = 0.682
Q
Overall
Years of regular smoking: AgeAccelDiff
Kruskal-Wallis test p = 0.398
R
Years of regular smoking: IEAA
Kruskal-Wallis test p = 0.262
SMK.Cat
S
HEI-2015, whole fruits: DNAmAge
Wilcoxon test p = 0.0707
T
Overall
HEI-2015, whole fruits: AgeAccelDiff
Wilcoxon test p = 0.019
U
HEI-2015, whole fruits: IEAA
Wilcoxon test p = 0.006
HEI2015.fruit.Cat
V
HEI-2015, vegetables: DNAmAge
Wilcoxon test p = 0.716
W
Overall
HEI-2015, vegetables: AgeAccelDiff
Wilcoxon test p = 0.159
X
HEI-2015, vegetables: IEAA
Wilcoxon test p = 0.368
HEI2015.veg.Cat

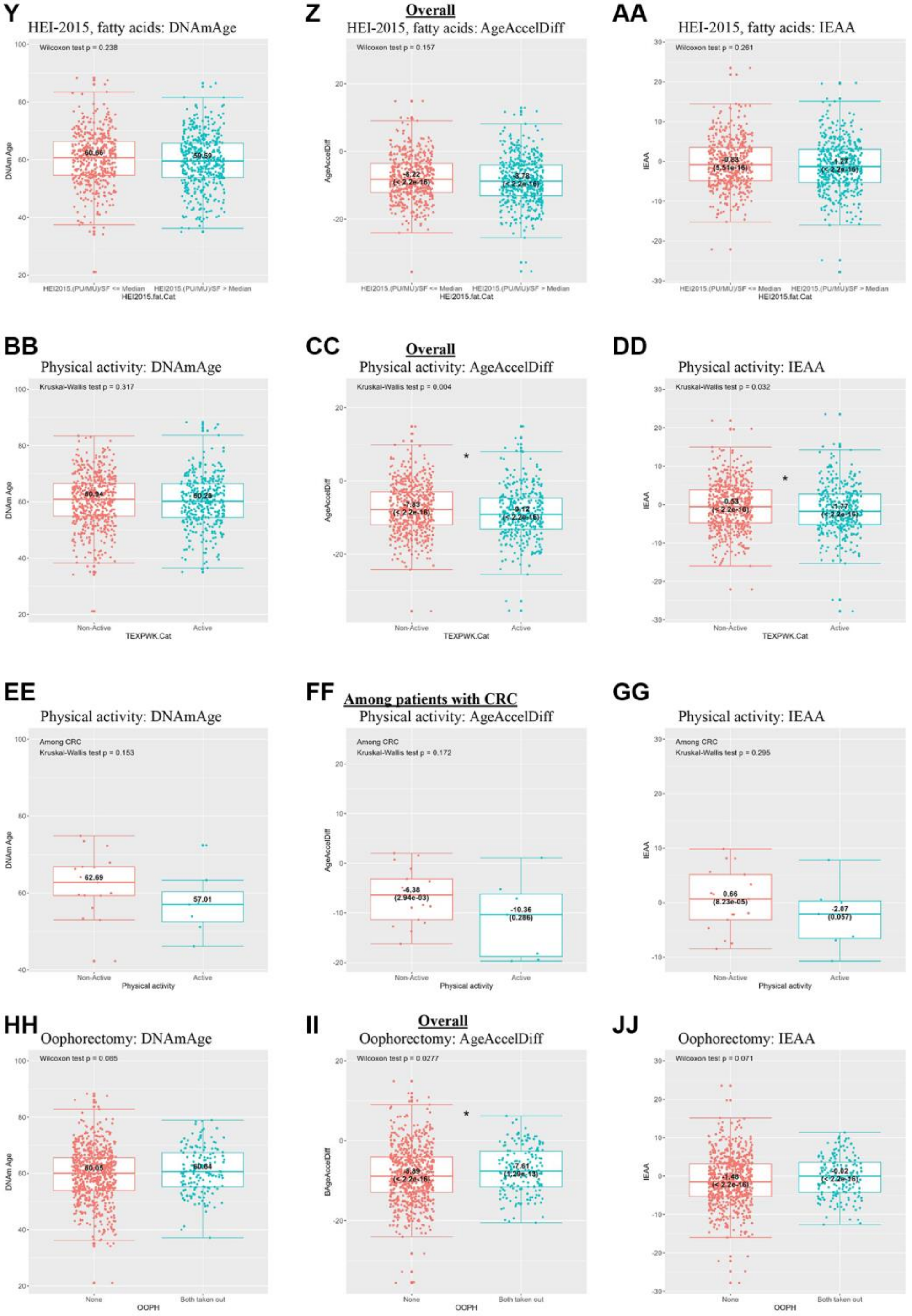
Y
HEI-2015, fatty acids: DNAmAge
Wilcoxon test p = 0.238
Z
Overall
HEI-2015, fatty acids: AgeAccelDiff
Wilcoxon test p = 0.157
AA
HEI-2015, fatty acids: IEAA
Wilcoxon test p = 0.261
HEI2015.fat.Cat
BB
Physical activity: DNAmAge
Kruskal-Wallis test p = 0.317
CC
Overall
Physical activity: AgeAccelDiff
Kruskal-Wallis test p = 0.004
DD
Physical activity: IEAA
Kruskal-Wallis test p = 0.032
Non-Active
Active
TEXPWK.Cat
EE
Physical activity: DNAmAge
Among CRC
Kruskal-Wallis test p = 0.153
62.69
57.01
FF
Among patients with CRC
Physical activity: AgeAccelDiff
Among CRC
Kruskal-Wallis test p = 0.172
-6.38
(2.94e-03)
-10.36
(0.286)
GG
Physical activity: IEAA
Among CRC
Kruskal-Wallis test p = 0.295
0.66
(8.23e-05)
-2.07
(0.057)
Physical activity
HH
Oophorectomy: DNAmAge
Wilcoxon test p = 0.085
II
Overall
Oophorectomy: AgeAccelDiff
Wilcoxon test p = 0.0277
JJ
Oophorectomy: IEAA
Wilcoxon test p = 0.071
None
Both taken out
OOPH

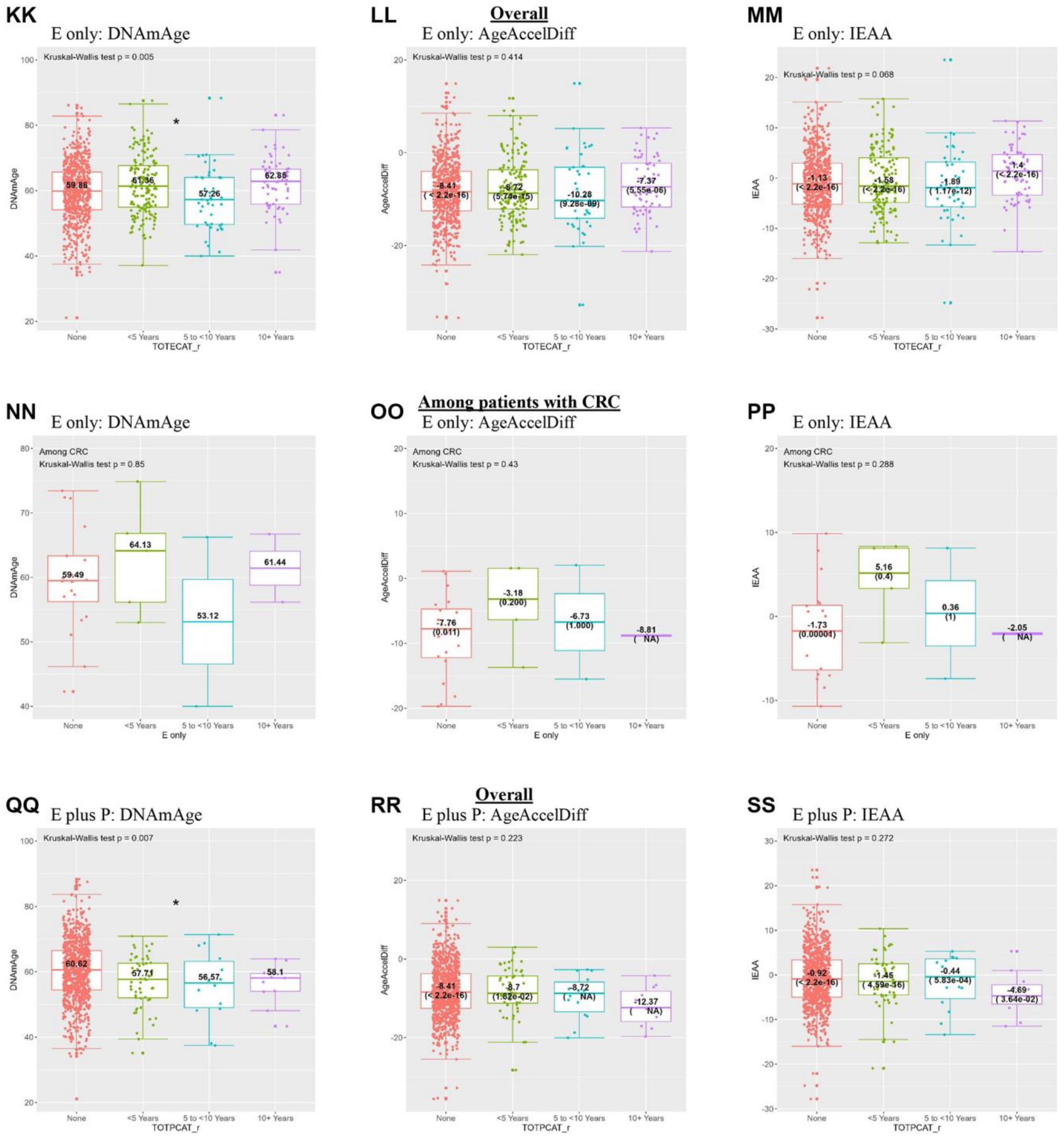

Supplementary Figure 3. Levine's clock: distribution of DNAmAge, AgeAccelDiff, and IEAA by selected CRC risk factors. Abbreviations: AgeAccelDiff: epigenetic age acceleration as departure of DNAmAge from chronologic age; BMI: body mass index; CRC: colorectal cancer; DNAmAge: DNA methylationbased marker of aging; E only: exogenous estrogen only; E plus P: E plus progestin; HEI-2015: Healthy Eating Index-2015; IEAA: intrinsic epigenetic age acceleration as residuals adjusted for cell composition; WHR: waist-to-hip ratio.

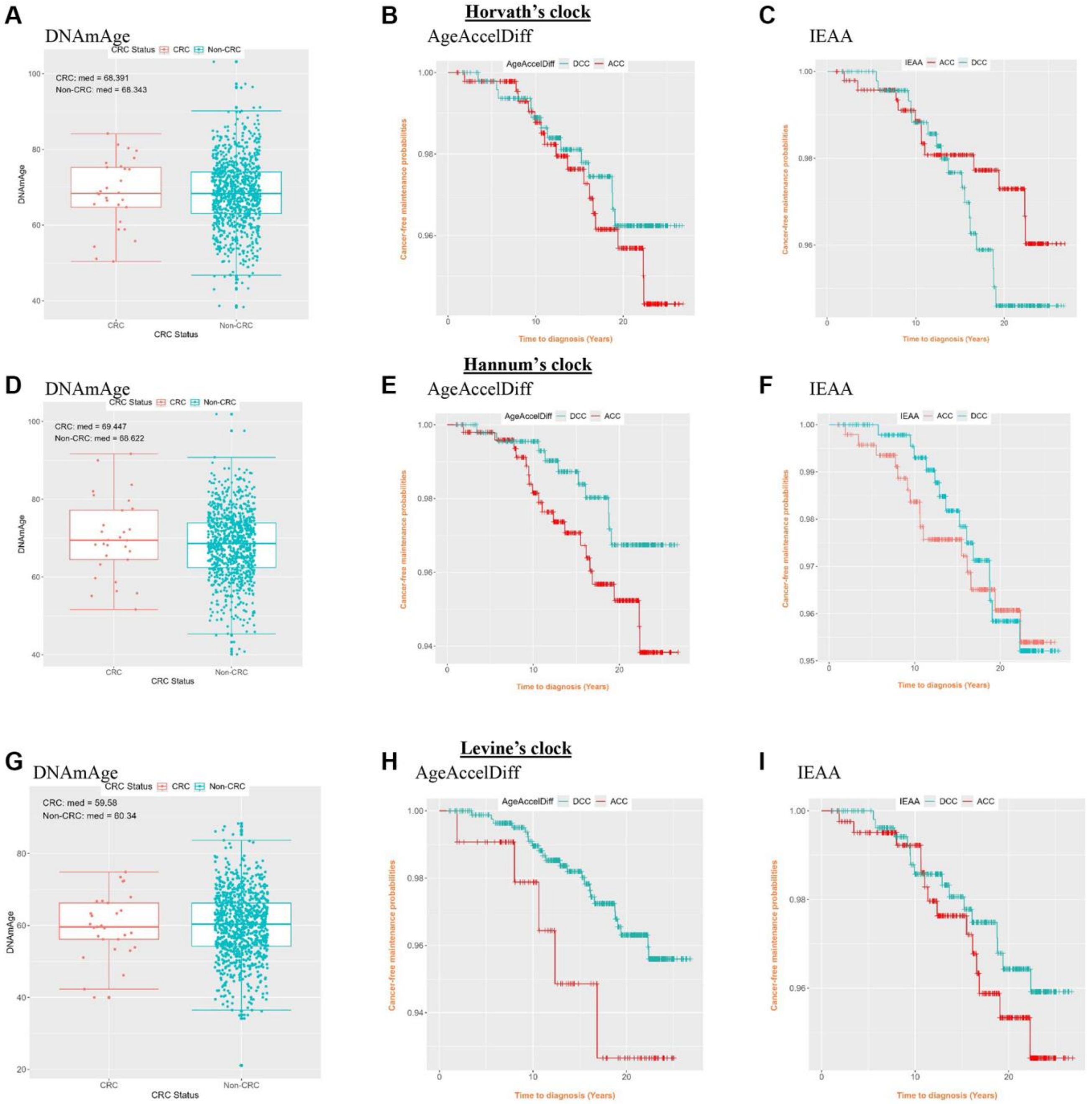

Supplementary Figure 4. Distribution of DNAmAge by CRC status and cancer-free probability curve of AgeAccelDiff and IEAA. Abbreviations: AgeAccelDiff: epigenetic age acceleration as departure of DNAmAge from chronologic age; ACC: accelerated age, i.e., positive difference of DNAm age from chronologic age; CRC: colorectal cancer; DNAmAge: DNA methylation-based marker of aging; DCC: decelerated age, i.e., negative difference of DNAm age from chronologic age; IEAA: intrinsic epigenetic age acceleration as residuals adjusted for cell composition.

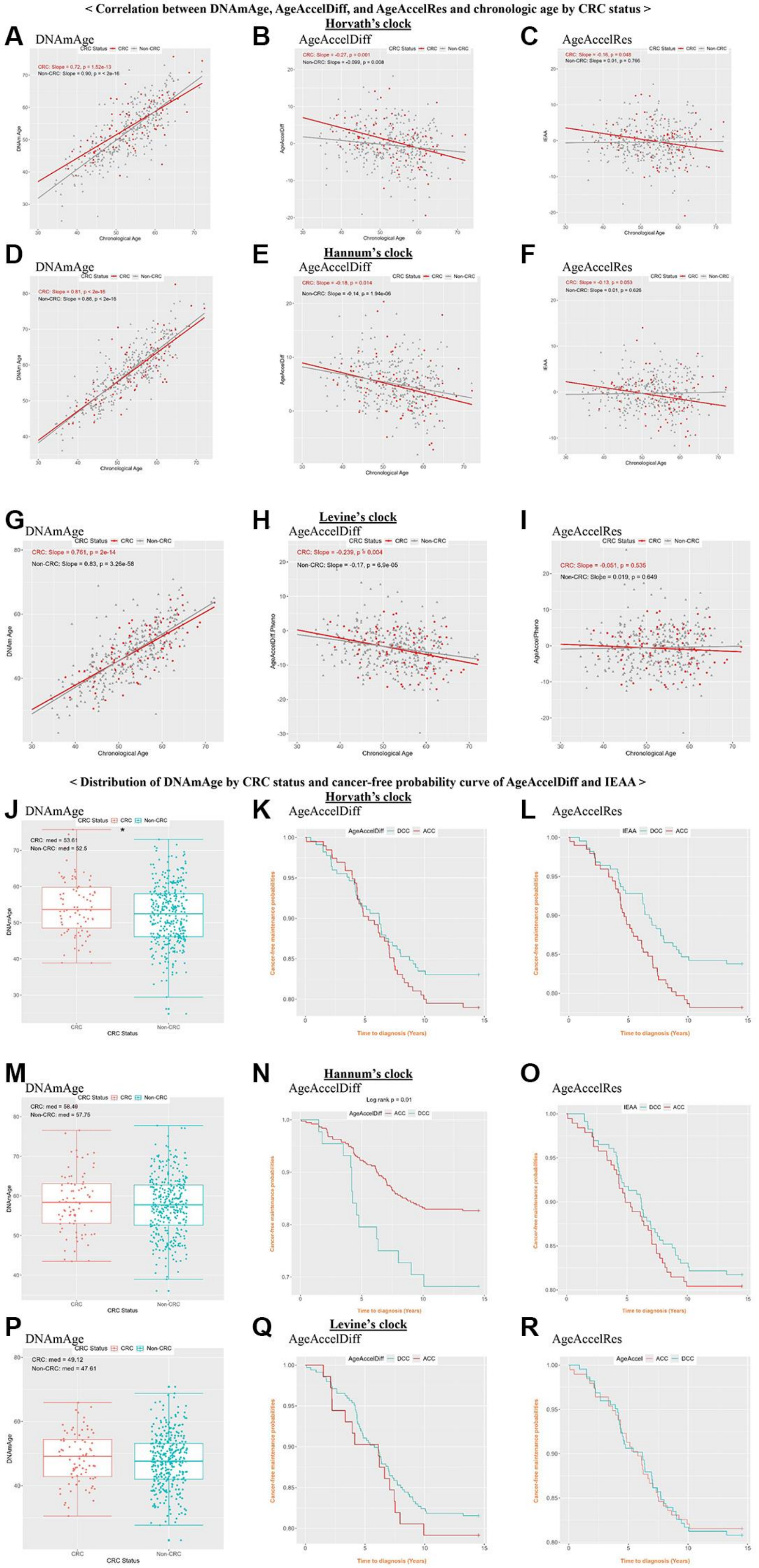

Supplementary Figure 5. GSE51032 women, validation tests. Abbreviations: AgeAccelDiff: epigenetic age acceleration as departure of DNAmAge from chronologic age; ACC: accelerated age, i.e., positive difference of DNAm age from chronologic age; CRC: colorectal cancer; DCC: decelerated age, i.e., negative difference of DNAm age from chronologic age; DNAmAge: DNA methylation-based marker of aging; IEAA, intrinsic epigenetic age acceleration as residuals adjusted for cell composition.

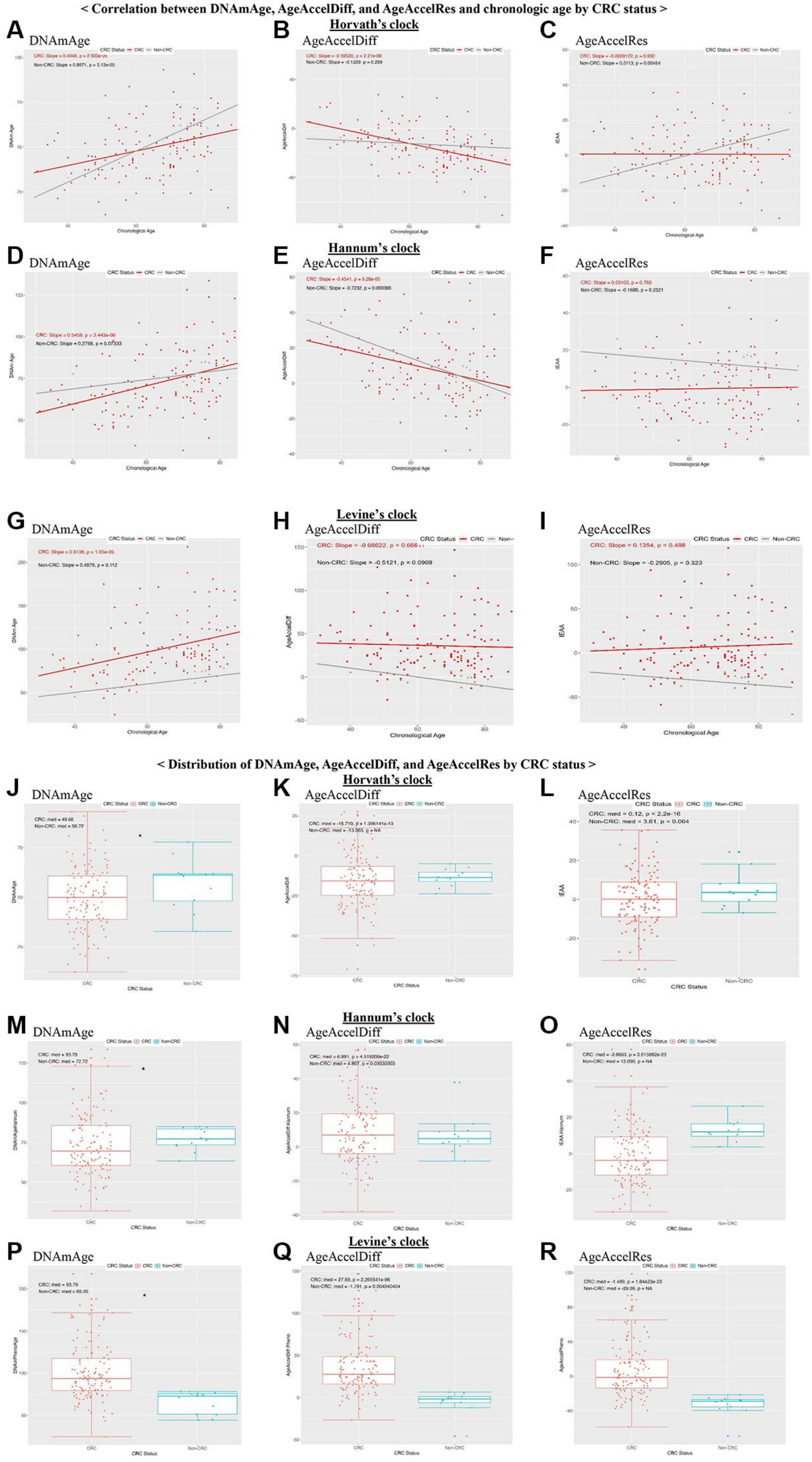

Supplementary Figure 6. TCGA women, validation tests. Abbreviations: AgeAccelDiff: epigenetic age acceleration as departure of DNAmAge from chronologic age; AgeAccelRes: epigenetic age acceleration as residuals by regressing DNAmAge on chronologic age; CRC: colorectal cancer; DNAmAge: DNA methylation-based marker of aging.

< Correlation between DNAmAge, AgeAccelDiff, and AgeAccelRes and chronologic age by CRC status >

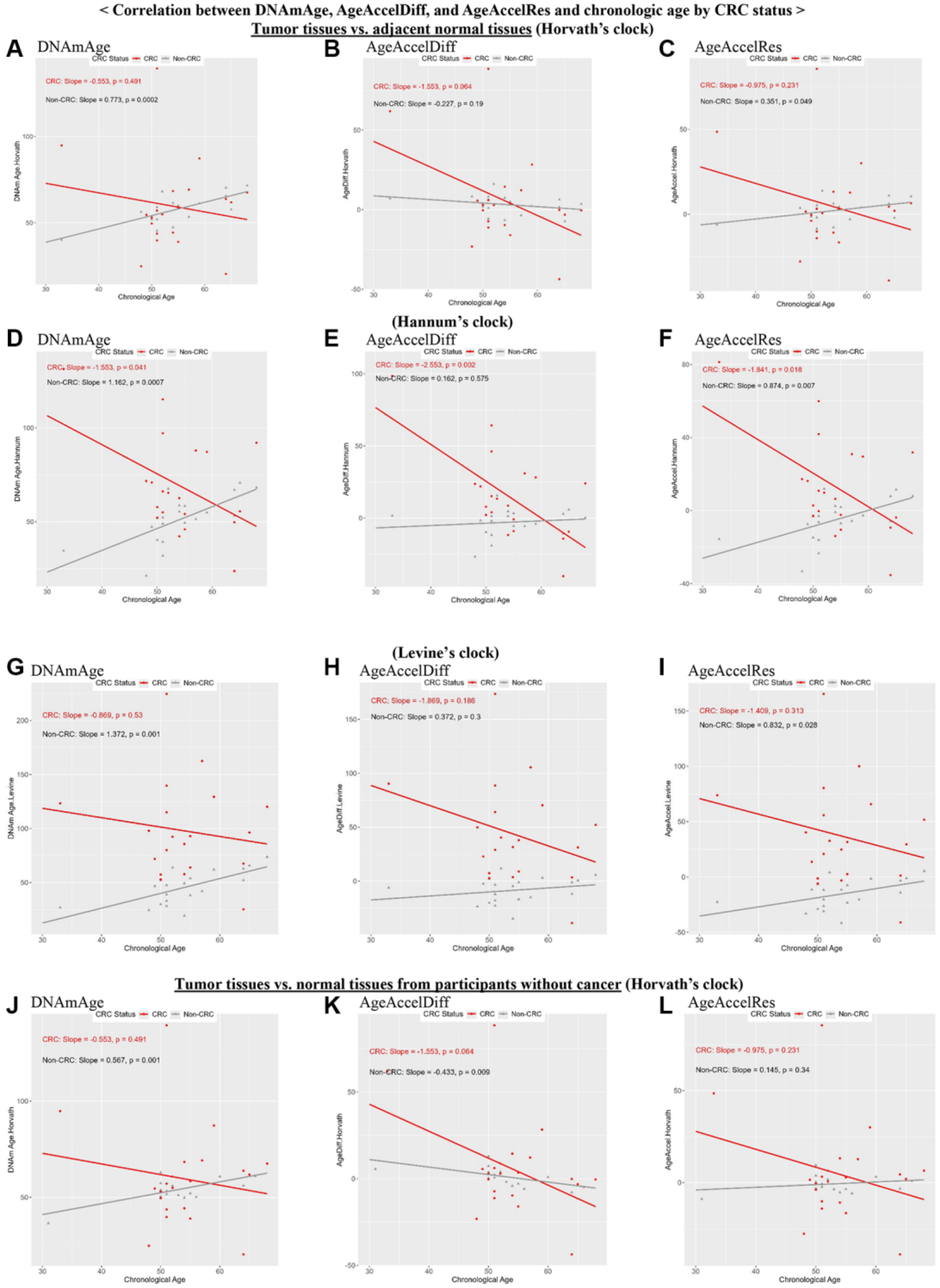

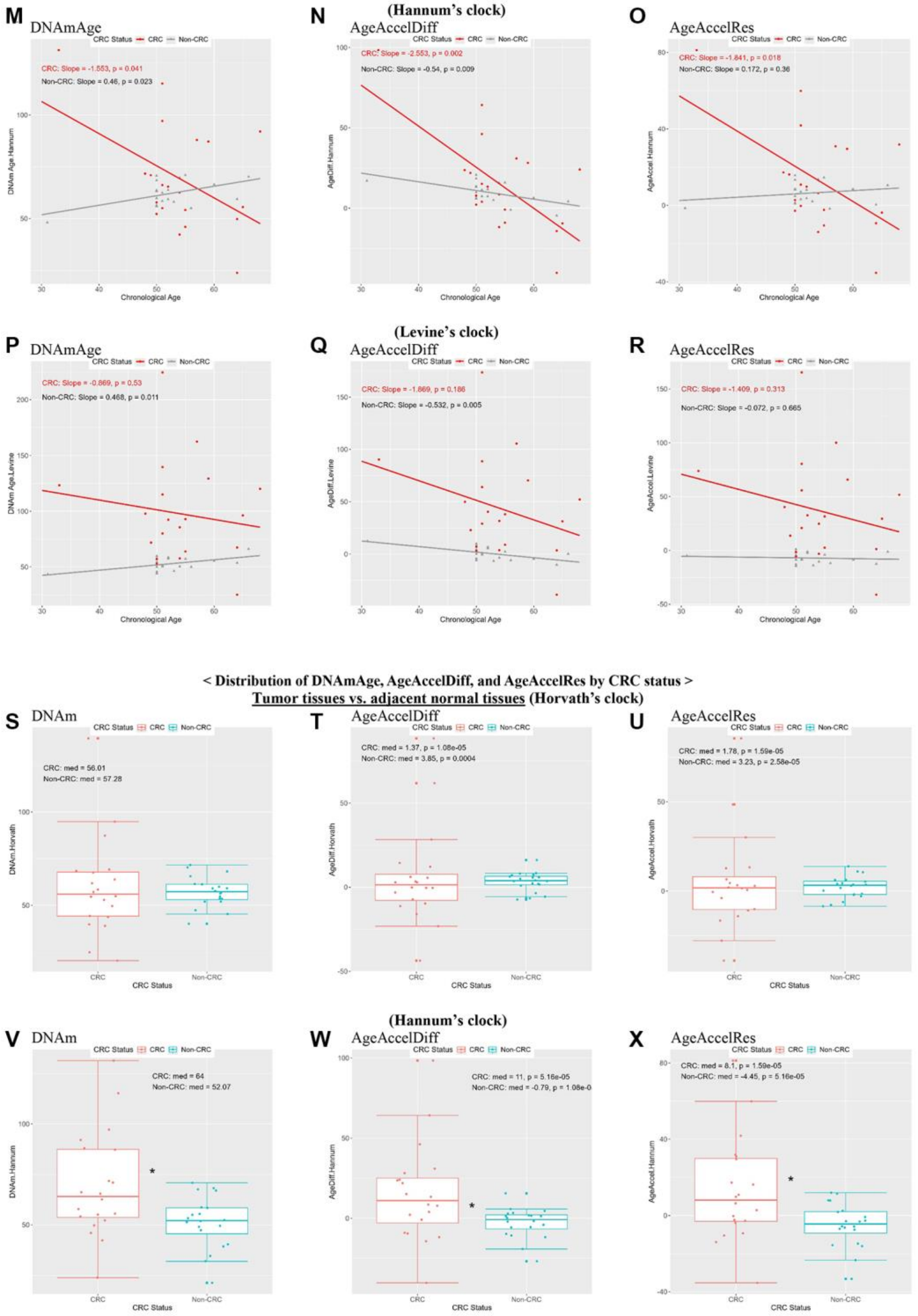
M DNAmAge
N (Hannum's clock) AgeAccelDiff
O AgeAccelRes
CRC: Slope = -1.553, p = 0.041
Non-CRC: Slope = 0.46, p = 0.023
CRC: Slope = -2.553, p = 0.002
Non-CRC: Slope = -0.54, p = 0.009
CRC: Slope = -1.841, p = 0.018
Non-CRC: Slope = 0.172, p = 0.36
P DNAmAge
Q (Levine's clock) AgeAccelDiff
R AgeAccelRes
CRC: Slope = -0.869, p = 0.53
Non-CRC: Slope = 0.468, p = 0.011
CRC: Slope = -1.869, p = 0.186
Non-CRC: Slope = -0.532, p = 0.005
CRC: Slope = -1.409, p = 0.313
Non-CRC: Slope = -0.072, p = 0.665
Chronological Age
CRC Status
< Distribution of DNAmAge, AgeAccelDiff, and AgeAccelRes by CRC status >
Tumor tissues vs. adjacent normal tissues (Horvath's clock)
S DNAm
T AgeAccelDiff
U AgeAccelRes
CRC: med = 56.01
Non-CRC: med = 57.28
CRC: med = 1.37, p = 1.08e-05
Non-CRC: med = 3.85, p = 0.0004
CRC: med = 1.78, p = 1.59e-05
Non-CRC: med = 3.23, p = 2.58e-05
V DNAm
W (Hannum's clock) AgeAccelDiff
X AgeAccelRes
CRC: med = 64
Non-CRC: med = 52.07
CRC: med = 11, p = 5.16e-05
Non-CRC: med = -0.79, p = 1.08e-0
CRC: med = 8.1, p = 1.59e-05
Non-CRC: med = -4.45, p = 5.16e-05
CRC
Non-CRC

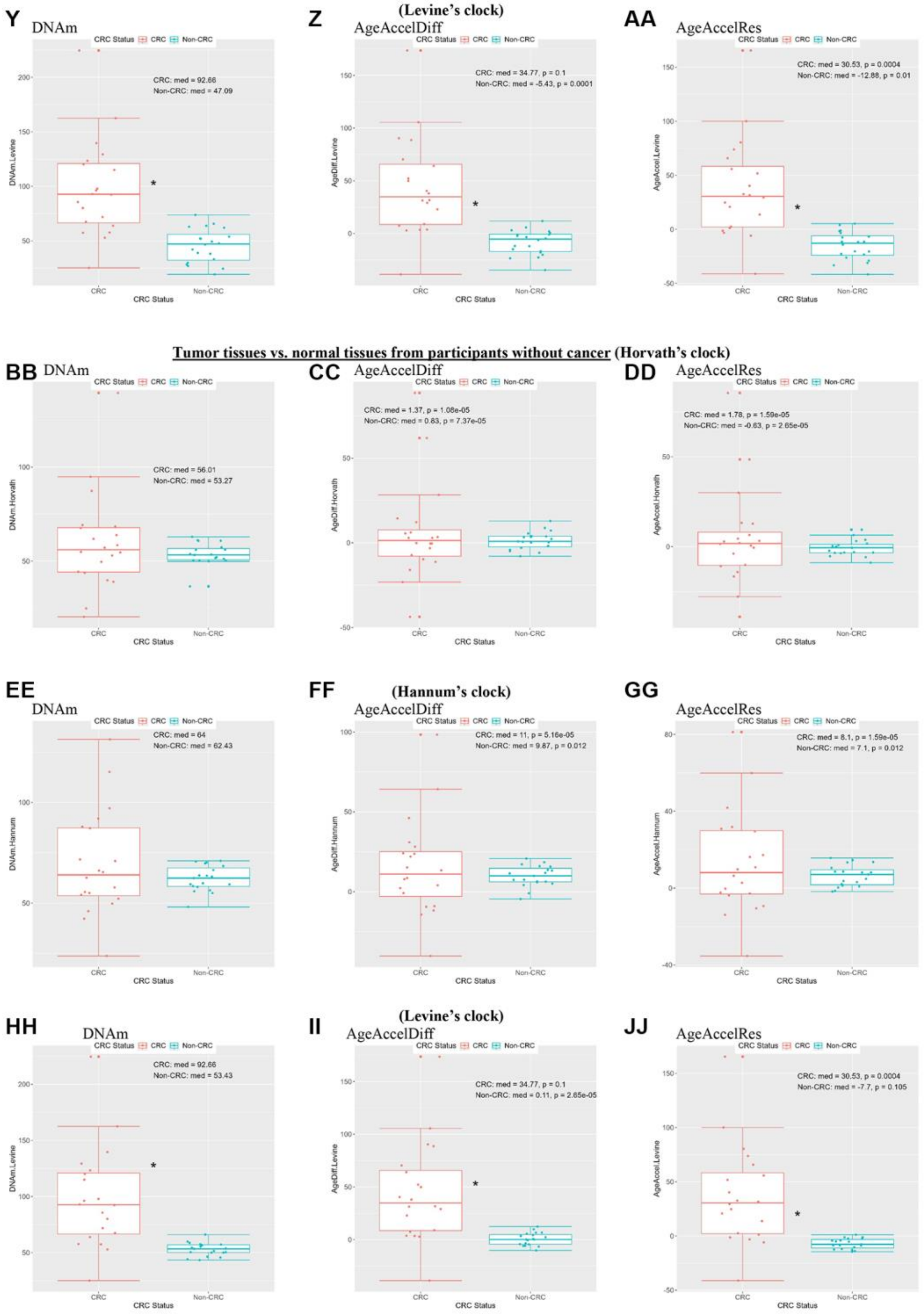

Supplementary Figure 7. GSE199057 women, validation tests. Abbreviations: AgeAccelDiff: epigenetic age acceleration as departure of DNAmAge from chronologic age; AgeAccelRes: epigenetic age acceleration as residuals by regressing DNAmAge on chronologic age; CRC: colorectal cancer; DNAmAge: DNA methylation-based marker of aging.
