# Supplementary material for: Epigenetic age and accelerated aging phenotypes: a tumor biomarker for predicting colorectal cancer
Source: Aging (Albany NY). 2025 Jul 7;17(7):1624–66. doi: 10.18632/aging.206276 (PMC12339025; doi:10.18632/aging.206276)
Supplement: Supplementary Tables [file aging-17-206276-s002.pdf]

SUPPLEMENTARY TABLES

Supplementary Table 1. Association of AgeAccelDiff and IEAA in Horvath's clock with selected CRC risk factors*; AgeAccelDiff and IEDAA were analyzed as binary outcomes (negatives vs. positives).

CRC risk factor	OR	95% CI	*P*
**A. AgeAccelDiff**			
BMI	**1.03**	**(1.01, 1.06)**	**0.006**
BMI** (normal weight vs. underweight, BMI <18.5)	1.86	(0.52, 7.43)	0.344
Overweight, BMI >25 and BMI <30	0.99	(0.71, 1.37)	0.941
Obesity, BMI >30 and BMI <40	**1.52**	**(1.10, 2.11)**	**0.012**
Extreme obesity, BMI >40	**2.61**	**(1.21, 5.98)**	**0.018**
**B. IEAA**			
BMI	**1.04**	**(1.01, 1.06)**	**0.002**
BMI** (normal weight vs. underweight, BMI <18.5)	1.30	(0.35, 4.78)	0.683
Overweight, BMI >25 and BMI <30	1.25	(0.90, 1.73)	0.188
Obesity, BMI >30 and BMI <40	**1.57**	**(1.13, 2.18)**	**0.007**
Extreme obesity, BMI >40	**3.18**	**(1.45, 7.53)**	**0.005**
Dietary alcohol intake (g/d)	**0.99**	**(0.97, 1.00)**	**0.046**
Dietary alcohol intake (g/d)¥ (<14g vs. >14g)	**0.64**	**(0.43, 0.95)**	**0.028**
Alcohol intake** (never vs. past drinker)	**1.68**	**(1.05, 2.69)**	**0.032**
<1 drink per month	1.46	(0.90, 2.38)	0.130
<1 drink per week	1.42	(0.89, 2.26)	0.140
1 to <7 drinks per week	1.33	(0.85, 2.08)	0.209
7+ drinks per week	0.88	(0.53, 1.47)	0.625

*Only factors having a *statistically significant* association with AgeAccelDiff are displayed. **Variables were further significant in a multiple regression model, adjusting for covariates (age, BMI, waist-to-hip ratio, type 2 diabetes, oophorectomy history, hormone replacement therapy, diet including whole fruits, vegetables, and fatty acids from Healthy Eating Index-2015, alcohol intake, years of regular smoking, and physical activity (except tested variable(s))). ¥Dietary alcohol intake (g/d) was dichotomized by 14 g as a moderate drink for women. Numbers in bold face are statistically significant. Abbreviations: AgeAccelDiff: epigenetic age acceleration measured as departure of DNAmAge from chronologic age; BMI: body mass index; CI: confidence interval; CRC: colorectal cancer; IEAA: intrinsic epigenetic age acceleration as residuals adjusted for cell composition; OR: odds ratio.

Supplementary Table 2. Association of DNAmAge, AgeAccelDiff, and IEAA in Hannum's clock with selected CRC risk factors*; AgeAccelDiff and IEAA were further analyzed as binary outcomes (negatives vs. positives).

CRC risk factor	Effect size	95% CI	*P*
**A. DNAm Age (Continuous outcomes)**			
Age**	**0.98**	**(0.91, 1.04)**	**2.72E-14**
BMI§ (normal weight vs. underweight, BMI <18.5)	0.37	(−3.57, 4.31)	0.854
Overweight, BMI >25 and BMI <30	0.24	(−0.90, 1.37)	0.684
Obesity, BMI >30 and BMI <40	0.53	(−0.70, 1.77)	0.395
Extreme obesity, BMI >40	**3.46**	**(0.76, 6.15)**	**0.012**
Waist-to-hip ratio	**9.50**	**(2.46, 16.54)**	**0.008**
Waist-to-hip ratio¥ (<0.85 vs. >0.85)	**1.17**	**(0.04, 2.30)**	**0.042**
Alcohol intake (never vs. past drinker)	−0.61	(−2.64, 1.42)	0.555
<1 drink per month	−2.05	(−4.15, 0.06)	0.057
<1 drink per week	−1.54	(−3.53, 0.46)	0.131
1 to <7 drinks per week	**−2.04**	**(−3.96, −0.12)**	**0.037**
7+ drinks per week	−1.78	(−3.98, 0.42)	0.112
Years of regular smoking (never vs. <5 years)	−0.24	(−2.07, 1.58)	0.793
5 to <20 years	**−1.99**	**(−3.81, −0.18)**	**0.032**
20 + years	**−1.81**	**(−3.17, −0.44)**	**0.010**
Healthy Eating Index-2015, whole fruits	**0.82**	**(0.42, 1.21)**	**4.86E-05**
Healthy Eating Index-2015, whole fruits¥ (<4.10 vs. >4.10)	**1.96**	**(0.76, 3.17)**	**0.001**
Exogenous estrogen only (never use vs. <5 years)	**1.70**	**(0.22, 3.18)**	**0.024**
5 to <10 years	−1.11	(−3.72, 1.49)	0.402
10 + years	1.47	(−0.73, 3.67)	0.190
Exogenous estrogen plus progestin (never use vs. <5 years)	**−2.96**	**(−5.29, −0.63)**	**0.013**
5 to <10 years	−4.15	(−8.71, 0.41)	0.074
10 + years	0.30	(−4.83, 5.44)	0.908
**Among only CRC patients**			
Alcohol intake (never vs. past drinker)	−0.64	(−12.51, 11.24)	0.912
<1 drink per month	−1.78	(−14.23, 10.68)	0.770
<1 drink per week	**22.73**	**(0.51, 44.95)**	**0.045**
1+ drinks per week	2.43	(−8.20, 13.06)	0.641
Years of regular smoking (never vs. <5 years)	**22.13**	**(2.30, 41.97)**	**0.030**
20 + years	−4.33	(−13.98, 5.31)	0.363
**B. AgeAccelDiff (Continuous outcomes)**			
BMI	**0.13**	**(0.06, 0.20)**	**0.0002**
BMI** (normal weight vs. underweight, BMI <18.5)	0.35	(−3.51, 4.20)	0.860
Overweight, BMI >25 and BMI <30	0.63	(−0.37, 1.62)	0.215
Obesity, BMI >30 and BMI <40	**1.40**	**(0.41, 2.39)**	**0.006**
Extreme obesity, BMI >40	**3.70**	**(1.43, 5.98)**	**0.001**
Waist-to-hip ratio	**6.29**	**(1.31, 11.28)**	**0.013**
Physical activity¥ (<10 MET vs. >10 MET)	**−0.93**	**(−1.75, −0.11)**	**0.027**
**Among only CRC patients**			
Alcohol intake (never vs. past drinker)	−1.29	(−8.87, 6.29)	0.728
<1 drink per month	0.61	(−7.34, 8.56)	0.874
<1 drink per week	**18.18**	**(4.00, 32.36)**	**0.014**
1+ drinks per week	0.31	(−6.31, 6.93)	0.924

Years of regular smoking (never vs. <5 years)	**18.71**	**(5.74, 31.67)**	**0.007**
20 + years	−0.80	(−7.10, 5.50)	0.795
**C. AgeAccelDiff (Binary outcomes)**	**OR**	**95% CI**	***P***
BMI	**1.03**	**(1.01, 1.06)**	**0.003**
BMI** (normal weight vs. underweight, BMI <18.5)	1.72	(0.48, 6.87)	0.408
Overweight, BMI >25 and BMI <30	1.13	(0.81, 1.56)	0.471
Obesity, BMI >30 and BMI <40	**1.44**	**(1.04, 2.00)**	**0.029**
Extreme obesity, BMI >40	**3.30**	**(1.48, 8.13)**	**0.005**
Waist-to-hip ratio	**6.35**	**(1.20, 35.28)**	**0.032**
Physical activity	**0.99**	**(0.98, 1.00)**	**0.021**
Physical activity**¥ (<10 MET vs. >10 MET)	**0.72**	**(0.55, 0.94)**	**0.017**
**D. IEAA (Continuous outcomes)**			
BMI	**0.05**	**(0.0001, 0.10)**	**0.050**
BMI (normal weight vs. underweight, BMI <18.5)	−0.01	(−2.68, 2.65)	0.991
Overweight, BMI >25 and BMI <30	0.47	(−0.22, 1.17)	0.183
Obesity, BMI >30 and BMI <40	**0.72**	**(0.03, 1.42)**	**0.042**
Extreme obesity, BMI >40	0.99	(−0.63, 2.61)	0.229
**Among only CRC patients**			
Alcohol intake (never vs. past drinker)	0.71	(−5.05, 6.46)	0.801
<1 drink per month	0.13	(−5.91, 6.17)	0.965
<1 drink per week	**14.05**	**(3.28, 24.82)**	**0.013**
1+ drinks per week	1.31	(−3.81, 6.43)	0.601
Years of regular smoking (never vs. <5 years)	**14.04**	**(4.90, 23.19)**	**0.004**
20 + years	1.11	(−3.34, 5.55)	0.612
**E. IEAA (Binary outcomes)**	**OR**	**95% CI**	***P***
BMI	**1.02**	**(1.00, 1.05)**	**0.038**
Waist-to-hip ratio	**5.66**	**(1.08, 31.13)**	**0.043**
Alcohol intake** (never vs. past drinker)	1.45	(0.91, 2.34)	0.120
<1 drink per month	**1.81**	**(1.11, 2.97)**	**0.018**
<1 drink per week	1.47	(0.92, 2.34)	0.105
1 to <7 drinks per week	1.35	(0.86, 2.11)	0.194
7+ drinks per week	1.52	(0.91, 2.54)	0.109
Healthy Eating Index-2015, whole fruits	**0.90**	**(0.82, 0.98)**	**0.021**
Healthy Eating Index-2015, whole fruits¥ (<4.10 vs. >4.10)	**0.75**	**(0.57, 1.00)**	**0.047**
Exogenous estrogen plus progestin** (never use vs. <5 years)	**0.55**	**(0.31, 0.95)**	**0.036**
5 to <10 years	1.32	(0.45, 4.02)	0.615
10 + years	1.73	(0.52, 6.63)	0.387

*Only factors having a *statistically significant* association with DNAmAge/AgeAccelDiff/IEAA are displayed. *Variables were further significant in a multiple regression model, adjusting for covariates (age, BMI, waist-to-hip ratio, type 2 diabetes, oophorectomy history, hormone replacement therapy, diet including whole fruits, vegetables, and fatty acids from Healthy Eating Index-2015, alcohol intake, years of regular smoking, and physical activity (except tested variable(s))). §Variable was significant only in a multiple regression model. ¥Waist-to-hip ratio was categorized using 0.85 as the cutoff, at which higher values fall into the viscerally obese range [84]; Healthy Eating Index-2015, whole fruits, was dichotomized by the mean, 4.10; Physical activity was estimated from recreational physical activity records combining walking and mild, moderate, and strenuous physical activity. Each activity was assigned a MET value corresponding to intensity and the total METJiouivweek per week was stratified into two groups, with 10 METs as the cutoff according to current American College of Sports Medicine and American Heart Association recommendations [63]. Numbers in bold face are statistically significant. Abbreviations: AgeAccelDiff: epigenetic age acceleration measured as departure of DNAmAge from chronologic age; BMI, body mass index; CI: confidence interval; CRC: colorectal cancer; DNAmAge: DNA methylation-based marker of aging; IEAA: intrinsic epigenetic age acceleration as residuals adjusted for cell composition; MET: metabolic equivalent; OR: odds ratio.

Supplementary Table 3. Association of DNAmAge, AgeAccelDiff, and IEAA in Levine's clock with selected CRC risk factors[*]; AgeAccelDiff and IEAA were further analyzed as binary outcomes (negatives vs. positives).

CRC risk factor	Effect size	95% CI	*P*
**A. DNAm Age (Continuous outcomes)**			
Age[**]	**0.98**	**(0.92, 1.05)**	**7.24E-13**
Type 2 diabetes	**2.56**	**(0.15, 4.98)**	**0.038**
BMI[§] (normal weight vs. underweight, BMI <18.5)	2.26	(−2.03, 6.54)	0.301
Overweight, BMI >25 and BMI <30	0.14	(−1.09, 1.38)	0.821
Obesity, BMI >30 and BMI <40	1.06	(−0.28, 2.40)	0.122
Extreme obesity, BMI >40	**4.96**	**(2.03, 7.88)**	**0.001**
Waist-to-hip ratio[**]	**15.66**	**(8.24, 23.08)**	**3.73E-05**
Waist-to-hip ratio[¥€] (<0.85 vs. >0.85)	**2.52**	**(1.33, 3.71)**	**3.55E-05**
Alcohol intake (never vs. past drinker)	−0.99	(−3.14, 1.16)	0.368
<1 drink per month	−1.39	(−3.62, 0.84)	0.221
<1 drink per week	−1.08	(−3.20, 1.03)	0.315
1 to <7 drinks per week	**−2.63**	**(−4.67, −0.60)**	**0.011**
7+ drinks per week	−1.33	(−3.66, 1.00)	0.261
Healthy Eating Index-2015, whole fruits	**0.73**	**(0.31, 1.14)**	**0.001**
Healthy Eating Index-2015, whole fruits[¥] (<4.10 vs. >4.10)	**1.55**	**(0.28, 2.83)**	**0.017**
Oophorectomy history (never vs. both ovary removal)	**1.64**	**(0.10, 3.18)**	**0.037**
Exogenous estrogen only (never use vs. <5 years)	**2.20**	**(0.65, 3.76)**	**0.006**
5+ years^	0.62	(−1.22, 2.46)	0.509
5 to <10 years	−2.04	(−4.80, 0.71)	0.145
10 + years	**2.46**	**(0.14, 4.79)**	**0.038**
Exogenous estrogen plus progestin (never use vs. <5 years)	**−3.50**	**(−5.97, −1.03)**	**0.005**
5+ years^	**−4.28**	**(−7.91, −0.65)**	**0.021**
5 to <10 years	−4.51	(−9.33, 0.31)	0.067
10 + years	−3.98	(−9.41, 1.45)	0.151
**B. AgeAccelDiff (Continuous outcomes)**			
Type 2 diabetes	**1.86**	**(0.08, 3.65)**	**0.041**
BMI	**0.20**	**(0.12, 0.27)**	**3.78E-07**
BMI[**] (normal weight vs. underweight, BMI <18.5)	1.65	(−2.58, 5.88)	0.444
Overweight, BMI >25 and BMI <30	0.84	(−0.25, 1.93)	0.132
Obesity, BMI >30 and BMI <40	**2.14**	**(1.05, 3.23)**	**0.0001**
Extreme obesity, BMI >40	**5.99**	**(3.49, 8.48)**	**2.82E-06**
Waist-to-hip ratio[**]	**12.45**	**(6.97, 17.93)**	**9.21E-06**
Waist-to-hip ratio[¥] (<0.85 vs. >0.85)	**2.08**	**(1.21, 2.96)**	**3.64E-06**
Healthy Eating Index-2015, whole fruits[¥] (<4.10 vs. >4.10)	**−1.06**	**(−2.01, −0.12)**	**0.027**
Healthy Eating Index-2015, vegetables[¥] (<4.23 vs. >4.23)	**−0.42**	**(−0.82, −0.02)**	**0.042**
Healthy Eating Index-2015, fatty acids[¥] (<4.20 vs. >4.20)	**−0.18**	**(−0.35, −0.02)**	**0.025**
Physical activity[¥€] (<10 MET vs. >10 MET)	**−1.31**	**(−2.22, −0.40)**	**0.005**
Oophorectomy history (never vs. both ovary removal)	**1.20**	**(0.07, 2.33)**	**0.038**
**C. AgeAccelDiff (Binary outcomes)**	**OR**	**95% CI**	***P***
Type 2 diabetes	**2.13**	**(1.05, 4.03)**	**0.027**
BMI	**1.07**	**(1.03, 1.10)**	**0.0001**
BMI (normal weight vs. underweight, BMI <18.5)	2.70	(0.39, 11.63)	0.226
Overweight, BMI >25 and BMI <30	1.06	(0.60, 1.91)	0.844
Obesity, BMI >30 and BMI <40	1.67	(0.98, 2.91)	0.063

Extreme obesity, BMI >40	**5.95**	**(2.48, 13.91)**	**4.40E-05**
Waist-to-hip ratio[**]	**120.77**	**(10.26, 1689.38)**	**0.0002**
Waist-to-hip ratio[¥] (<0.85 vs. >0.85)	**1.97**	**(1.31, 2.95)**	**0.001**
Physical activity[¥] (<10 MET vs. >10 MET)	**0.56**	**(0.35, 0.87)**	**0.011**
**D. IEAA (Continuous outcomes)**			
Age[§]	**0.08**	**(0.004, 0.15)**	**0.038**
BMI[**]	**0.18**	**(0.11, 0.25)**	**3.60E-07**
BMI (normal weight vs. underweight, BMI <18.5)	0.39	(−3.51, 4.28)	0.845
Overweight, BMI >25 and BMI <30	0.90	(−0.11, 1.90)	0.080
Obesity, BMI >30 and BMI <40	**1.99**	**(0.99, 2.99)**	**0.0001**
Extreme obesity, BMI >40	**5.49**	**(3.20, 7.79)**	**3.07E-06**
Waist-to-hip ratio[**]	**12.35**	**(7.31, 17.39)**	**1.77E-06**
Waist-to-hip ratio[¥] (<0.85 vs. >0.85)	**1.99**	**(1.18, 2.80)**	**1.51E-06**
Years of regular smoking (never vs. <5 years)	0.27	(−1.06, 1.60)	0.691
5 to <20 years	0.40	(−0.92, 1.73)	0.550
20 + years	**1.04**	**(0.04, 2.04)**	**0.041**
Healthy Eating Index-2015, whole fruits	**−0.31**	**(−0.59, −0.03)**	**0.033**
Healthy Eating Index-2015, whole fruits[**¥] (<4.10 vs. >4.10)	**−1.23**	**(−2.09, −0.36)**	**0.006**
Physical activity[¥] (<10 MET vs. >10 MET)	**−0.93**	**(−1.78, −0.09)**	**0.030**
**Among only CRC patients**			
Exogenous estrogen only (never use vs. <5 years)	**5.88**	**(0.13, 11.62)**	**0.045**
5 to <10 years	1.88	(−6.64, 10.40)	0.654
10 + years	−0.54	(−9.05, 7.98)	0.898
**E. IEAA (Binary outcomes)**	**OR**	**95% CI**	***P***
Age[§]	**1.04**	**(1.01, 1.06)**	**0.007**
BMI	**1.06**	**(1.04, 1.09)**	**3.25E-07**
BMI[**] (normal weight vs. underweight, BMI <18.5)	0.90	(0.19, 3.32)	0.878
Overweight, BMI >25 and BMI <30	**1.57**	**(1.12, 2.21)**	**0.010**
Obesity, BMI >30 and BMI <40	**2.06**	**(1.47, 2.89)**	**2.88E-05**
Extreme obesity, BMI >40	**8.73**	**(3.67, 24.23)**	**4.75E-06**
Waist-to-hip ratio	**32.67**	**(5.77, 194.55)**	**0.0001**
Waist-to-hip ratio[¥] (<0.85 vs. >0.85)	**1.79**	**(1.37, 2.33)**	**1.62E-05**
Years of regular smoking[§] (never vs. <5 years)	1.30	(0.80, 2.10)	0.284
5 to <20 years	1.32	(0.80, 2.18)	0.272
20 + years	**1.53**	**(1.05, 2.23)**	**0.026**
Healthy Eating Index-2015, whole fruits	**0.88**	**(0.80, 0.96)**	**0.004**
Healthy Eating Index-2015, whole fruits[**¥] (<4.10 vs. >4.10)	**0.65**	**(0.49, 0.85)**	**0.002**
Physical activity[¥] (<10 MET vs. >10 MET)	**0.76**	**(0.58, 1.00)**	**0.049**

[*]Only factors having a *statistically significant* association with DNAmAge/AgeAccelDiff/IEAA are displayed. [**]Variables were further significant in a multiple regression model, adjusting for covariates (age, BMI, waist-to-hip ratio, type 2 diabetes, oophorectomy history, hormone replacement therapy, diet including whole fruits, vegetables, and fatty acids from Healthy Eating Index-2015, alcohol intake, years of regular smoking, and physical activity (except tested variable(s))). [§]Variables were significant only in a multiple regression model. [¥]Waist-to-hip ratio was categorized using 0.85 as the cutoff, at which higher values fall into the viscerally obese range [84]; Healthy Eating Index-2015, whole fruits, vegetables, and fatty acids, was dichotomized by the mean, 4.10, the mean, 4.23, and the mean, 4.20, respectively; physical activity was estimated from recreational physical activity records combining walking and mild, moderate, and strenuous physical activity. Each activity was assigned a MET value corresponding to intensity and the total MET hours week per week was stratified into two groups, with 10 METs as the cutoff according to current American College of Sports Medicine and American Heart Association recommendations [63]. [€]Variables were further significant among CRC patients only as follows: waist-to-hip ratio (<0.85 vs.

>0.85) in DNAm Age: effect size = 7.2, 95% CI (0.98, 13.4); physical activity (<10 MET vs. >10 MET) in AgeAccelDiff continuous outcomes: effect size = −0.52, 95% CI (−0.84, −0.21). [¶]Five years and longer duration of use of exogenous estrogen only and the estrogen plus progestin was further stratified into 5 to <10 years and 10 + years. Numbers in bold face are statistically significant. Abbreviations: AgeAccelDiff: epigenetic age acceleration measured as departure of DNAmAge from chronologic age; BMI: body mass index; CI: confidence interval; CRC: colorectal cancer; DNAmAge: DNA methylation-based marker of aging; IEAA: intrinsic epigenetic age acceleration as residuals adjusted for cell composition; MET: metabolic equivalent; OR: odds ratio.

Supplementary Table 4. Multiple Cox regression for DNAmAge, AgeAccelDiff, and IEAA predicting CRC development within 15 years.

DNAm clock	15 years			5–15 years[¥]		
	HR[†]	95% CI	*P*	HR[†]	95% CI	*P*
**Horvath's clock**						
DNAmAge[*]	**1.07**	**(1.01, 1.15)**	**0.033**			
DNAmAge, 10-year interval	**3.54**	**(1.68, 7.46)**	**0.001**	**2.77**	**(1.29, 5.97)**	**0.009**
AgeAccelDiff[*]	1.06	(0.98, 1.16)	0.148			
AgeAccelDiff, 10-year interval	0.54	(0.20, 1.42)	0.211			
AgeAccelDiff, ACC vs. DCC	**4.26**	**(1.29, 14.12)**	**0.018**			
IEAA[*]	1.05	(0.93, 1.19)	0.437			
IEAA, 10-year interval	2.36	(0.72, 7.79)	0.157			
IEAA, ACC vs. DCC	2.09	(0.63, 6.88)	0.226			
**Hannum's clock**						
DNAmAge[*]	**1.23**	**(1.12, 1.36)**	**1.20E-05**	**1.20**	**(1.09, 1.32)**	**0.0001**
DNAmAge, 10-year interval	**4.33**	**(2.02, 9.29)**	**0.0002**	**3.75**	**(1.74, 8.06)**	**0.001**
AgeAccelDiff[*]	**1.20**	**(1.06, 1.36)**	**0.005**			
AgeAccelDiff, 10-year interval	**8.02**	**(2.34, 27.51)**	**0.001**	**5.34**	**(1.55, 18.42)**	**0.008**
AgeAccelDiff, ACC vs. DCC	**8.77**	**(2.50, 30.83)**	**0.001**	**5.61**	**(1.56, 20.19)**	**0.008**
IEAA[*]	1.15	(0.99, 1.33)	0.070			
IEAA, 10-year interval	3.01	(0.90, 10.04)	0.073			
IEAA, ACC vs. DCC	3.05	(0.91, 10.17)	0.070			
**Levine's clock**						
DNAmAge[*]	**1.09**	**(1.00, 1.18)**	**0.038**			
DNAmAge, 10-year interval	1.81	(0.93, 3.55)	0.083			
AgeAccelDiff[*]	1.07	(0.98, 1.16)	0.140			
AgeAccelDiff, 10-year interval	**3.43**	**(1.66, 7.08)**	**0.001**			
AgeAccelDiff, ACC vs. DCC	**10.52**	**(2.68, 41.34)**	**0.001**			
IEAA[*]	1.07	(0.98, 1.18)	0.145			
IEAA, 10-year interval	1.47	(0.54, 3.98)	0.448			
IEAA, ACC vs. DCC	**3.29**	**(0.99, 10.91)**	**0.051**			

[*]DNAmAge, AgeAccelDiff, and IEAA were each analyzed as a continuous variable. [†]HR adjusted for all covariates (age, body mass index, waist-to-hip ratio, type 2 diabetes, oophorectomy history, hormone replacement therapy, diet including whole fruits, vegetables, and fatty acids from Healthy Eating Index-2015, alcohol intake, years of regular smoking, and physical activity). Numbers in bold face are statistically significant. [¥]Only results with statistically significance were presented. Abbreviations: ACC: accelerated age (positive deviation of DNAm age from chronological age); AgeAccelDiff: epigenetic age acceleration measured as departure of DNAmAge from chronologic age; CI: confidence interval; CRC: colorectal cancer; DCC: decelerated age (negative deviation of DNAm age from chronological age); DNAmAge: DNA methylation-based marker of aging; HR: hazard ratio; IEAA: intrinsic epigenetic age acceleration as residuals adjusted for cell composition.

Supplementary Table 5. GSE51032 women, validation tests: Cox regression for DNAmAge, AgeAccelDiff, and IEAA predicting CRC development§.

DNAm clock	HR†	95% CI	*P*
**Horvath's clock¥**			
DNAmAge*	**1.03**	**(1.00, 1.06)**	**0.029**
DNAmAge, 10-year interval	**1.32**	**(1.03, 1.70)**	**0.028**
AgeAccelDiff*	1.03	(0.98, 1.07)	0.236
AgeAccelDiff, 10-year interval	1.23	(0.85, 1.79)	0.271
IEAA*	1.01	(0.96, 1.06)	0.806
IEAA, 10-year interval	1.38	(0.94, 2.01)	0.097
**Hannum's clock**			
DNAmAge*	1.01	(0.99, 1.04)	0.335
DNAmAge, 10-year interval	1.15	(0.87, 1.52)	0.324
AgeAccelDiff*	0.97	(0.92, 1.02)	0.231
AgeAccelDiff, 10-year interval	0.88	(0.54, 1.43)	0.599
IEAA*	0.96	(0.90, 1.02)	0.219
IEAA, 10-year interval	1.12	(0.74, 1.70)	0.593
**Levine's clock**			
DNAmAge*	1.01	(0.99, 1.04)	0.368
DNAmAge, 10-year interval	1.06	(0.82, 1.36)	0.660
AgeAccelDiff*	0.98	(0.95, 1.02)	0.423
AgeAccelDiff, 10-year interval	0.83	(0.59, 1.17)	0.290
IEAA*	0.99	(0.95, 1.03)	0.678
IEAA, 10-year interval	0.78	(0.55, 1.10)	0.151

§GSE51032 women were followed for their CRC development during 15 years. ¥Horvath's clock: DNAmAge confined within 5 to 15 years, per a 1-year increase, HR = 1.04; 95% CI (1.00, 1.08); $p = 0.037$. *DNAmAge, AgeAccelDiff, and IEAA were each analyzed as a continuous variable. †HRs obtained from univariate analysis. Numbers in bold face are statistically significant. Abbreviations: AgeAccelDiff: epigenetic age acceleration measured as departure of DNAmAge from chronologic age; CI: confidence interval; CRC: colorectal cancer; DNAmAge: DNA methylation-based marker of aging; HR: hazard ratio; IEAA: intrinsic epigenetic age acceleration as residuals adjusted for cell composition.

Supplementary Table 6. TCGA women, validation tests: logistic regression for DNAmAge, AgeAccelDiff, and AgeAccelRes in association with CRC.

DNAm clock	OR†	95% CI	*P*
**Horvath's clock**			
DNAmAge*	0.97	(0.93, 1.01)	0.128
DNAmAge, 10-year interval	0.71	(0.48, 1.04)	0.083
AgeAccelDiff*	0.99	(0.96, 1.03)	0.766
AgeAccelDiff, 10-year interval	0.96	(0.67, 1.39)	0.840
AgeAccelRes*	1.01	(0.93, 1.11)	0.745
AgeAccelRes, 10-year interval	0.81	(0.52, 1.26)	0.349
**Hannum's clock**			
DNAmAge*	0.99	(0.96, 1.02)	0.471
DNAmAge, 10-year interval	0.89	(0.66, 1.22)	0.437
AgeAccelDiff*	1.01	(0.97, 1.04)	0.758
AgeAccelDiff, 10-year interval	1.06	(0.76, 1.51)	0.742
AgeAccelRes*	**0.95**	**(0.91, 0.99)**	**0.009**

AgeAccelRes, 10-year interval	**0.59**	**(0.39, 0.85)**	**0.006**
**Levine's clock[¥]**			
DNAmAge*	**1.07**	**(1.03, 1.11)**	**0.0004**
DNAmAge, 10-year interval	**1.88**	**(1.36, 2.80)**	**0.001**
AgeAccelDiff*	**1.13**	**(1.07, 1.22)**	**0.0003**
AgeAccelDiff, 10-year interval	**3.27**	**(1.92, 6.83)**	**0.0002**
AgeAccelRes*	**1.12**	**(1.07, 1.21)**	**0.0003**
AgeAccelRes, 10-year interval	**3.39**	**(1.93, 7.52)**	**0.0004**

*DNAmAge, AgeAccelDiff, and AgeAccelRes were each analyzed as a continuous variable. ¥Levine's clock: AgeAccelDiff, ACC, compared with DCC, OR = 31.5; 95% CI (8.23, 141.62); p = 1.29E-06. †ORs obtained from univariate analysis. Numbers in bold face are statistically significant. Abbreviations: ACC: accelerated age (positive deviation of DNAm age from age); AgeAccelDiff: epigenetic age acceleration measured as departure of DNAmAge from chronologic age; AgeAccelRes: epigenetic age acceleration as residuals by regressing DNAmAge on chronologic age; CI: confidence interval; CRC: colorectal cancer; DCC: decelerated age (negative deviation of DNAm age from age); DNAmAge: DNA methylation-based marker of aging; OR: odds ratio; TCGA: The Cancer Genomic Atlas.

Supplementary Table 7. GSE199057 women, validation tests: logistic regression for DNAmAge, AgeAccelDiff, and AgeAccelRes in association with CRC.

DNAm clock	Tumor tissues vs. adjacent normal tissues			Tumor tissues vs. normal tissues from patients without cancer		
	OR†	95% CI	*P*	OR†	95% CI	*P*
**Horvath's clock**						
DNAmAge*	1.01	(0.97, 1.05)	0.701	1.02	(0.98, 1.07)	0.351
DNAmAge, 10-year interval	1.03	(0.72, 1.49)	0.863	1.10	(0.77, 1.65)	0.615
AgeAccelDiff*	1.01	(0.97, 1.04)	0.717	1.01	(0.98, 1.05)	0.525
AgeAccelDiff, 10-year interval	1.04	(0.75, 1.47)	0.812	1.07	(0.78, 1.54)	0.687
AgeAccelRes*	1.01	(0.97, 1.05)	0.703	1.02	(0.98, 1.06)	0.415
AgeAccelRes, 10-year interval	1.06	(0.75, 1.53)	0.740	1.19	(0.84, 1.87)	0.363
**Hannum's clock**						
DNAmAge*	**1.05**	**(1.01, 1.11)**	**0.023**	1.02	(0.99, 1.07)	0.259
DNAmAge, 10-year interval	**1.74**	**(1.17, 3.01)**	**0.020**	1.27	(0.90, 1.94)	0.212
AgeAccelDiff*	**1.05**	**(1.01, 1.11)**	**0.034**	1.01	(0.98, 1.05)	0.447
AgeAccelDiff, 10-year interval	**1.55**	**(1.08, 2.55)**	**0.044**	1.13	(0.85, 1.61)	0.423
AgeAccelRes*	**1.06**	**(1.01, 1.12)**	**0.025**	1.02	(0.99, 1.06)	0.306
AgeAccelRes, 10-year interval	**1.61**	**(1.11, 2.68)**	**0.031**	1.17	(0.85, 1.72)	0.370
**Levine's clock[¥]**						
DNAmAge*	**1.09**	**(1.04, 1.17)**	**0.003**	**1.11**	**(1.05, 1.23)**	**0.010**
DNAmAge, 10-year interval	**2.15**	**(1.44, 4.04)**	**0.003**	**2.75**	**(1.54, 7.94)**	**0.010**
AgeAccelDiff*	**1.10**	**(1.04, 1.20)**	**0.005**	**1.09**	**(1.04, 1.18)**	**0.009**
AgeAccelDiff, 10-year interval	**2.55**	**(1.54, 5.93)**	**0.004**	**2.32**	**(1.42, 5.29)**	**0.009**
AgeAccelRes*	**1.10**	**(1.04, 1.19)**	**0.004**	**1.10**	**(1.04, 1.22)**	**0.011**
AgeAccelRes, 10-year interval	**2.41**	**(1.51, 5.17)**	**0.004**	**2.46**	**(1.46, 5.99)**	**0.009**

*DNAmAge, AgeAccelDiff, and AgeAccelRes were each analyzed as a continuous variable. ¥Levine's clock: AgeAccelDiff, ACC, compared with DCC, OR = 13.82; 95% CI (2.13, 274.56); p = 0.020. †ORs obtained from univariate analysis. Numbers in bold face are statistically significant. Abbreviations: ACC: accelerated age (positive deviation of DNAm age from age); AgeAccelDiff: epigenetic age acceleration measured as departure of DNAmAge from chronologic age; AgeAccelRes: epigenetic age acceleration as residuals by regressing DNAmAge on chronologic age; CI: confidence interval; CRC: colorectal cancer; DCC: decelerated age (negative deviation of DNAm age from age); DNAmAge: DNA methylation-based marker of aging; OR: odds ratio.
